# Mesenchymal stem cell-derived exosomes in myocardial infarction repair: therapeutic potential and scaffold-based delivery strategies

**DOI:** 10.3389/fphar.2026.1762630

**Published:** 2026-02-05

**Authors:** Vajiheh Azimian Zavareh, Negar Eslampoor, Sanaz Panahi-Alanagh, Latifeh Malekmohammad, Agata Stanek

**Affiliations:** 1 Department of Plant and Animal Biology, Faculty of Biological Science and Technology, University of Isfahan, Isfahan, Iran; 2 Department of Internal Medicine, Metabolic Diseases and Angiology, Faculty of Health Sciences in Katowice, Medical University of Silesia, Katowice, Poland

**Keywords:** apoptosis, cardiac repair, exosome, inflammation, mesenchymal stem cells, myocardial infarction, scaffold

## Abstract

Myocardial infarction (MI) remains a leading cause of global mortality, with current therapeutic modalities offering limited capacity for complete myocardial tissue regeneration. Advances in regenerative medicine have introduced stem cell-based approaches, among which mesenchymal stem cells (MSCs) have garnered significant scientific curiosity due to their multipotent differentiation potential and favorable safety profile. However, evidence suggests that the primary therapeutic effects of MSCs are mediated through their paracrine secretion of bioactive factors, notably exosomes. These MSC-derived exosomes (MSC-Exos) can modulate key aspects of cardiac repair, such as enhancing angiogenesis, preventing apoptosis, and alleviating inflammation by transferring genetic material such as miRNAs, proteins, and lipids and by activating molecular pathways critical to cardiac repair. Numerous studies as well as preclinical and clinical trials are currently investigating MSC-Exos for tissue regeneration. This review critically examines the biological characteristics and underlying mechanisms of MSC-Exos in myocardial repair, with particular focus on cell sources such as bone marrow-derived MSCs (BMMSCs), adipose-derived MSCs (ADSCs), and human umbilical cord MSCs (HUCMSCs), and evaluates their roles from multiple perspectives. Moreover, this review emphasizes innovative delivery approaches, including hydrogel-based systems, aimed at maximizing therapeutic effectiveness and accelerating translational potential. The integration of scaffold technologies and exosome engineering holds substantial promise for translating this cell-free approach into effective clinical treatments, presenting MSC-Exos as a transformative strategy with the potential to markedly improve outcomes in MI.

## Graphical Abstract

**Figure:**
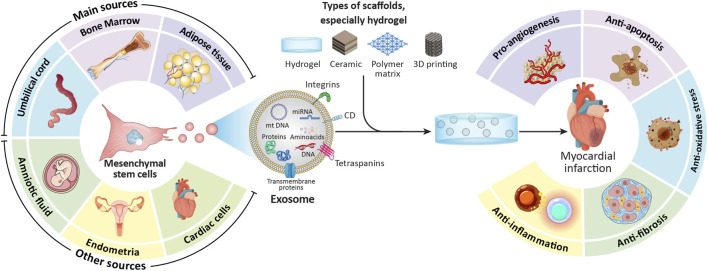

## 1 Introduction

Myocardial Infarction (MI) is a common condition and a leading cause of mortality worldwide. Following MI, normal physiological functions, cardiomyocyte proliferation, signaling pathways, and phenotypic characteristics are disrupted. Moreover, MI is associated with fibrotic alterations, activation of microRNAs (miRNAs), long noncoding RNAs (lncRNAs), and inflammatory responses that affect cardiomyocyte function. (Tzahor and Poss, 2017; Sheng et al., 2019). These processes contribute to reduced myocardial contractility and electrophysiological disturbance (Janse and Kleber, 1981).

Currently, procedures such as percutaneous transluminal coronary angioplasty, diagnostic cardiac catheterization, and bypass surgery are commonly utilized for the rapid diagnosis and treatment of MI. Despite these interventions, the 5-year mortality rate following MI remains elevated across all age groups, as these treatments do not effectively restore function in permanently damaged myocardial tissue (Virani et al., 2020). In this context, regenerative medicine, particularly cell therapy, has emerged as a viable alternative approach.

Various cell types are utilized in cardiac repair, including embryonic stem cells (ESCs), induced pluripotent stem cells (iPSCs), multipotent and unipotent adult stem cells (notably mesenchymal stem cells [MSCs]), cardiosphere-derived cells (CDCs), and endothelial progenitor cells (EPCs) (Shiba et al., 2009; Wang et al., 2013b; Kawaguchi and Nakanishi, 2022; Amjad et al., 2020). MSCs are multipotent adult stem cells that can be extracted from various sources, such as bone marrow, adipose tissue, and the umbilical cord (Suzuki et al., 2017). MSCs are particularly notable for their multilineage differentiation potential and the ease of their isolation; transplantation of these cells has demonstrated effectiveness in reducing myocardial damage and improving cardiac function following MI (Martínez et al., 2006). However, the benefits of MSC transplantation are likely not solely due to direct differentiation into cardiac tissue, as most cells do not persist in the heart (McGinley et al., 2013). Additionally, cell transplantation carries risks, including immune rejection, embolism, calcification or ossification at the infarct site, and arrhythmias (Zangi et al., 2009; Cui et al., 2015; Breitbach et al., 2007; Madonna et al., 2016). Therefore, it is essential to explore new strategies to enhance the efficacy of stem cell therapy (Beliën et al., 2022). Current therapeutic strategies focus on enhancing endothelial function, facilitating neovascularization, and preventing cardiomyocyte death to maintain cardiomyocyte contractility (Riaud et al., 2020).

MSCs are often evaluated for their ability to produce regenerative exosomes, which are recognized for their safety and effectiveness (Adamiak and Sahoo, 2018). A growing body of evidence demonstrates that the cardiac reparative benefits of MSCs are largely mediated by paracrine effects, especially the release of extracellular vesicles (EVs) (Chang et al., 2021; Dong et al., 2012; Harrell et al., 2019; Zhang et al., 2022). Hence, one of the most effective strategies for treating and repairing the heart after MI is the application of mesenchymal stem cell-derived secretions and exosomes. This approach serves as an alternative to direct stem cell transplantation, with stem cell-derived exosomes regarded as a promising treatment for MI (Huang et al., 2019). EVs constitute a varied category of nano-sized particles released by cells, and they are primarily categorized according to their dimensions. This classification includes exosomes (30–150 nm), microvesicles (100–1,000 nm), and apoptotic bodies (100–5,000 nm) (da Costa et al., 2021; Jeppesen et al., 2023). Exosomes are released by, and taken up by, nearly all cell types throughout the body (Yang et al., 2015). They transfer diverse genetic materials, including proteins, lipids, and RNAs, related to their origin and function. Exosomes can migrate from their source cells to different regions of the body *via* systemic circulation, enabling efficient delivery of their cargo (Pegtel et al., 2010; Skog et al., 2008; Kalluri and LeBleu, 2020). It seems that the therapeutic advantages of MSCs are largely attributed to their exosomes (Eleuteri and Fierabracci, 2019). The use of exosomes derived from human MSCs was among the initial studies exploring the paracrine mechanisms involved in cardiac repair (Zheng et al., 2022b). MSC-Exos present a cell-free alternative to mesenchymal stem cell transplantation, characterized by elevated safety profiles, absence of immune responses, and the capability to traverse biological barriers, thus preserving paracrine advantages without the inherent risks associated with live cell therapies (Zhao et al., 2019b). Numerous studies have demonstrated that MSC-Exos mitigate MI through various mechanisms, including promoting angiogenesis, preventing apoptosis, reducing fibrosis, and combating oxidative stress (Zou et al., 2019; Zheng et al., 2022a; Yin et al., 2023). Exosomes emanating from mesenchymal stem cells appear to modulate energy metabolic pathways within cardiac tissue regeneration, potentially assuming a critical function in efficacious myocardial repair (Yadav et al., 2024b). Investigative findings indicate that these exosomes may attenuate autophagic flux within compromised cardiac myocytes, which could potentially augment cardiac functionality and reduce the dimensions of infarcted regions (Charles et al., 2020). MSC-Exos are instrumental in cardiac protection by reducing oxidative stress, enhancing adenosine triphosphate (ATP) and nicotinamide adenine dinucleotide (reduced form) (NADH) production, regulating inflammation, and activating the PI3K/Akt pathway, which helps preserve left ventricular function after ischemia-reperfusion (I/R) injury (Khan et al., 2013). They also decrease infarct size in porcine models of I/R injury and reduce myocardial autophagy and cellular death after MI or I/R injury (Timmers et al., 2008; Jiang et al., 2018). Additionally, the therapeutic effects of MSC-Exos are mediated by their contained miRNAs, which regulate essential pathways for cardiac regeneration. For example, administration of miR-146a in a mouse model of MI leads to a decrease in infarct size and an improvement in cardiac function, whereas its absence results in no improvement (Jeevanantham et al., 2012). Empirical data suggest that MSC-Exos exert inhibitory effects on inflammation, decrease apoptotic events, and facilitate cardiac remodeling, corroborated by both preclinical and clinical investigations (Rayat Pisheh and Sani, 2025). It appears plausible that MSC-Exos may affect macrophage polarization through the action of microRNAs such as miR-21-5p (Shen and He, 2021; Qian et al., 2024). Innovations projected for the years 2024–2025, including inhalational exosome therapy (SCENT therapy), offer non-invasive, precise myocardial delivery and improved functional outcomes in animal models of myocardial infarction (Magadum, 2025).

The efficient delivery of MSC-Exo to target tissues remains challenging due to their short half-life, estimated at ∼2–30 min, with most exosomes cleared from circulation within 5 minutes (Zhao et al., 2024). This rapid removal complicates efficient localization and sustained therapeutic levels. To address this challenge, several preclinical strategies have been explored, such as embedding exosomes in hydrogels, incorporating them into scaffolds or microneedle patches, and engineering targeted delivery systems, all aiming to improve retention and efficacy (Zhao et al., 2023). The successful utilization of exosomes at the infarct site frequently depends on hydrogel systems. Recent advances in hydrogel technologies, including electrospun nanogels, phase separation, and polymer engineering, offer substantial potential for progression in regenerative cardiology (Zhou and Jin, 2023; Martin et al., 2022; Yang et al., 2022a). Methodologies employing three-dimensional scaffolds may synergistically incorporate exosomes with stem cells to enhance the efficacy of cardiac repair by a factor of twenty to forty (Gu et al., 2021). Hydrogels that integrate exosomes possess the potential to ameliorate cardiac architecture and functionality, thereby positioning themselves as a promising approach for cardiac regeneration (Yan et al., 2024a). Natural polymers exhibit substantial efficacy in the stabilization of exosomes and their targeted delivery to the injured locale, thereby augmenting therapeutic effectiveness while simultaneously minimizing adverse repercussions (Sun et al., 2025). The engineering of exosomes with cardioprotective microRNAs such as miR-302 or the utilization of conductive hydrogels may enhance their retention and mitigate macrophage-mediated clearance (Lipchina et al., 2011; Yan et al., 2024a).

Although investigations into hydrogel-based exosome delivery to the myocardium remain limited, research interest in this area is steadily increasing. Several recent reviews have broadly discussed MSC-derived exosomes in cardiovascular and cardiac diseases; an MI-focused synthesis that explicitly integrates exosome biology with scaffold-based delivery is still lacking (Rayat Pisheh and Sani, 2025; Hassanzadeh et al., 2024). Here, we specifically address myocardial infarction repair by critically comparing source-dependent MSC-Exos effects (BMMSC-Exos, ADSC-Exos, and HUCMSC-Exos) and systematically evaluating scaffold-enabled delivery strategies developed to mitigate rapid exosome clearance and improve myocardial retention. Beyond hydrogel platforms, we highlight advanced delivery formats such as cardiac patches and microneedle-based systems and discuss how these biomaterials can be engineered to enable sustained release and enhanced therapeutic efficacy in MI models. To provide a comprehensive perspective, we first examine the properties of MSC-derived exosomes and their mechanisms of action in cardiac tissue repair. We then review relevant studies to evaluate their therapeutic potential, and finally highlight scaffold-based biotherapeutic approaches designed to enhance the efficacy of exosome therapies in MI.

## 2 Exosome

Intercellular communication is a fundamental characteristic of multicellular organisms, occurring either through direct cell-to-cell communication or *via* the delivery of released biomolecules (He et al., 2024). Over the past 2 years, an additional mechanism mediated by EVs has been recognized as a pivotal mode of intercellular signaling. Traditional cell-cell interactions involve growth factors, chemokines, soluble factors, cytokines, and extracellular matrix components (Li et al., 2023b). Recent advances have demonstrated that cells also communicate through the release and uptake of membrane-bound EVs. These vesicles are secreted into the extracellular environment by a variety of mammalian cell types under both physiological and pathological conditions. Notably, while EV production is a normal part of cellular function, elevated EV levels have been documented in various disease contexts, including infectious and cardiovascular diseases (Kisielewska et al., 2024). Various cell types, including stem cells, adipocytes, B and T lymphocytes, dendritic cells, platelets, mast cells, neurons, endothelial cells and, epithelial cells participate in EV release, underscoring their broad significance in intercellular communication (Nail et al., 2023). EVs represent a diverse category of biological entities, encompassing microparticles, exosomes, ectosomes, microvesicles, oncosomes, and apoptotic bodies (Ma et al., 2025).

Exosomes are nano-sized membranous vesicles (30–100 nm), emanate from multivesicular bodies (MVBs) and are released into the extracellular space *via* the fusion of the plasma membrane. They are defined by their 5′-nucleotidase activity and diverse physiological activities, and they are notably recognized for their distinctive cup-shaped appearance. Exosomes possess a distinctive lipid bilayer, with an average thickness of around 5 nm (Lobb et al., 2015). They are distinguished from other EVs, such as extruded microvesicles or ectosomes (100–1,000 nm) (Cocucci et al., 2009) and apoptotic bodies produced during apoptosis (1–5 μm) (Cocucci and Meldolesi, 2015). Key determinants of exosome biology include size, origin (endosomal *versus* plasma membrane), specific surface markers, and their molecular cargo, all of which influence intercellular transport (Edgar, 2016; Matsumoto et al., 2020). Exosomes facilitate the transfer of a diverse set of molecular components—proteins, lipids, and nucleic acids—to recipient cells. Upon uptake of this cargo, recipient cells mount context-dependent responses (Simons and Raposo, 2009; Théry et al., 2018; Laulagnier et al., 2004; Keller et al., 2006; Mathivanan et al., 2010). Common exosomal cargo includes proteins: platelet-derived growth factor receptor, lactadherin, a range of transmembrane proteins, lysosome-associated membrane protein-2B, membrane transport and fusion proteins including annexins, flotillins, GTPases, heat shock proteins (HSPs), tetraspanins, and proteins associated with multivesicular body biogenesis, lipid-related proteins, and phospholipases (Alvarez-Erviti et al., 2011; Cooper et al., 2014; Conde-Vancells et al., 2008; Subra et al., 2010). Nucleic acids: deoxyribonucleic acid (DNA) and various ribonucleic acids (RNA), such as messenger RNA (mRNA) and miRNA (Perez-Moreno et al., 2003; Jenjaroenpun et al., 2013). Exosomes are characterized by a variety of surface markers such as tetraspanin family (CD9, CD63, CD81), Hsp60, Hsp70, Hsp90, membrane transport proteins (Rab GTPases, annexins), biogenesis-related proteins (endosomal sorting complex required for transport [ESCRT] family, Alix, tumor susceptibility gene 101 [TSG101]), and metabolic enzymes (glyceraldehyde-3-phosphate dehydrogenase [GAPDH], adenosine triphosphatase [ATPase], phosphoglycerate kinase 1 [PGK1]), which enable them to target of recipient cells, allowing exosomes to function as tissue-specific delivery vehicles (Witwer et al., 2019; Buzás et al., 2018; Lötvall et al., 2014; Antimisiaris et al., 2018).

Exosome isolation employs multiple methods that leverage specific exosome characteristics, including density, surface proteins, morphology, and dimensions. These approaches encompass size-based separation, differential ultracentrifugation, microfluidics, immunoaffinity capture, and exosome precipitation. Among these, ultracentrifugation is widely regarded as the gold standard and benchmark for exosomes (Pavani et al., 2018).

Exosomes are derived from a diverse range of cellular origins, including embryonic stem cells ESCs, iPSCs, EPCs, cancer stem cells (CSCs), CDCs, and both multipotent and unipotent adult stem cell (ASC) lineages (notably MSCs) (Balbi and Vassalli, 2020; Dilsiz, 2025; Aminzadeh et al., 2018). MSCs are frequently evaluated for their potential to generate regenerative exosomes, a capacity attributed to their established safety and efficacy (Adamiak and Sahoo, 2018). Building on the significance of MSC-Exos in tissue regeneration, the next section will explore the characteristics and therapeutic potential of MSC-Exos specifically in MI.

### 2.1 MSC and their derived exosomes the equations should be inserted in editable format from the equation editor

MSCs can be extracted from a wide variety of tissues and organs, such as bone marrow, adipose tissue, umbilical cord and cord blood, placenta, tendons, skin, salivary glands, skeletal muscle, synovial membranes, endometrium, amniotic membrane, amniotic fluid, peripheral blood, menstrual blood, dental pulp, and periodontal ligaments (Mushahary et al., 2018; Lan et al., 2021; Deo et al., 2022; Yin et al., 2023). In clinical trials, MSCs are predominantly sourced from adipose tissue, umbilical cord blood and bone marrow (Xu et al., 2017). MSCs express CD105, CD73, and CD90 but do not express CD45, CD34, CD14, CD11b, CD79α, CD19, or HLA-DR, and have the *in vitro* capacity to differentiate into adipocytes, osteoblasts, and chondrocytes (Hoang et al., 2019). These cells secrete a variety of cytokines, including vascular endothelial growth factor (VEGF) and pigment epithelial-derived factor (Ju et al., 2018). MSCs play an important role in cell therapy for many diseases. Preclinical studies utilizing rat and porcine models of MI have demonstrated that MSCs can significantly decrease the size of the infarction, restore myocardial contractility, and enhance the function and structure of damaged hearts through the combined effects of angiogenesis and myogenesis (Tang et al., 2006; Yang et al., 2011). Although several clinical trials have shown the efficacy of MSC therapy, other studies have reported no significant benefits from MSC treatment (Li et al., 2023a; Telukuntla et al., 2013). The variability in therapeutic outcomes associated with MSCs may be affected by variables including the technique of cell procurement, the transplantation process, cell survival post-transplantation, inadequate homing capabilities, and the high dosage required to sustain therapeutic effects (Joladarashi and Kishore, 2022; Yu et al., 2020).

MSC-Exos, similar to exosomes from other cell types, can mimic and replicate MSC biological functions, offering a safer, non-cell-based therapeutic alternative (Lai et al., 2013). MSC-Exos are popular in regenerative medicine and tissue regeneration [44], because of their multipotency and self-renewal properties. MSC-Exos are currently a viable treatment for tissue regeneration in various primary organs. Exosomes can address cardiovascular disease, enhance bone remodeling, and reverse liver fibrosis, and they have demonstrated promising regenerative effects across various models such as MI, kidney injury, and neurological injury (Bellin et al., 2019; Chen et al., 2019; Xie et al., 2017b). They have regenerative properties through several mechanisms: Inflammation and apoptosis are reduced, while proliferation and angiogenesis are promoted. Identifying the optimal combination of cargo loading and culture components will facilitate greater specialization and effective targeting of exosomes, thereby enhancing regeneration and clinical translatability. At present, numerous clinical trials are investigating the use of MSC-Exos for regenerative tissue; here, we will focus on their application in MI and myocardial defects.

## 3 Mechanisms and therapeutic potential of exosomes in myocardial infarction

One of the leading causes of mortality worldwide is cardiovascular disease. MI occurs when a blood clot blocks the flow of blood to the heart. Acute MI triggers myocardial cell death due to prolonged ischemia, illustrating its significant contribution to mortality in coronary artery disease. Following cardiac injury, there is a notable decrease in blood flow to the heart, leading to cardiomyocyte apoptosis or necrosis (Huang et al., 2023). This results in irreversible depletion of cardiomyocytes, remodeling of the left ventricle, and ultimately heart failure, as adult cardiac cells lack the capacity for regeneration after ischemic events. The prognosis of MI is significantly influenced by the extent of irreversible cardiomyocyte loss and the development of scar tissue (Leancă et al., 2022). Furthermore, dysregulation of immune pathways, inadequate suppression of post-infarction inflammation, spatially regulated suppression of the inflammatory response, and excessive fibrosis can adversely affect cardiac remodeling and contribute to the progression of heart failure (Hu et al., 2024). In the context of I/R injury, several pathological events occur, including endothelial inflammation, oxidative stress, and myocardial cell death (Liu et al., 2024). Additionally, reduced cardiac angiogenesis can hinder the restoration processes and reestablishment of standard cardiac function (Gladka et al., 2021).

Current studies have demonstrated that different types of exosomes are released by various cell types, including bone marrow mesenchymal stem cells (BMMSCs), adipose-derived mesenchymal stem cells (ADMSCs), and serum exosomes from coronary, particularly those secreted by cardiac coronary endothelial cells in response to myocardial ischemia, can mitigate myocardial injury. These exosomes exhibit properties that are anti-apoptotic, antioxidant, anti-inflammatory, anti-fibrotic, and pro-angiogenic properties.

Below are several processes affected by ventricular heart tissue damage, including programmed cell death (PCD), autophagy, inflammation, and angiogenesis.

### 3.1 PCD

Studies show that PCD pathways are activated in response to ischemia or hypoxia and are major contributors to heart failure (Zhang et al., 2020). Accumulating evidence indicates that MSCExos can impede PCD and fibrosis, stimulate angiogenesis, and improve the ischemic myocardium microenvironment (Sun et al., 2021; Bian et al., 2014). Notably, human ESC-derived MSC-Exos have been shown to reduce myocardial fibrosis and improve cardiac function for the first time (Lai et al., 2010). PCD encompasses several forms, including apoptosis, autophagy, pyroptosis, and ferroptosis. MSC-Exos may protect the myocardium against hypoxic and ischemic damage by inhibition of these processes (Navickas et al., 2016).

### 3.2 Autophagy

Autophagy is a process whereby lysosomes degrade organelles and other cellular components to remove unnecessary or damaged elements (Maejima et al., 2023). Recent investigations have indicated that a certain level of autophagy is beneficial for maintaining cardiomyocyte structure and function, with enhanced autophagy conferring cardioprotection during myocardial hypoxia and ischemia (Sciarretta et al., 2018).

### 3.3 Inflammation

Inflammation is a distinct stage of the cardiac repair process after MI and can be mitigated by exosomes (Ma et al., 2017b). Effective management of post-MI inflammation is crucial to reduce infarct size and improve clinical outcomes. Prolonged or excessive inflammation can drive adverse remodeling of the left ventricle and is associated with poorer prognosis (Ong et al., 2018). As repair progresses, the inflammatory response wanes, allowing most fibroblasts to differentiate into myofibroblasts, which adopt an anti-inflammatory phenotype and contribute to extracellular matrix (ECM) synthesis, thereby supporting myocardial contractility and structural/functional integrity (Guo et al., 2021). During the initial inflammatory phase, cardiac fibroblasts (CFs) may polarize toward a pro-inflammatory phenotype and contribute to ECM degradation (Shinde and Frangogiannis, 2014). At this stage, differentiation of CFs into myofibroblasts is inhibited. However, after dead cells are cleared, the process transitions to a proliferative stage, marked by a reduction in the inflammatory response. The rise in myocardial fibroblasts later in inflammation may aid repair by reducing myocardial (Ma et al., 2017b). Although the exact mechanism remains to be fully elucidated, MSC-Exos exhibit potent immunosuppressive and anti-inflammatory effects by modulating the myocardial microenvironment (Xiong et al., 2021). After MI, ATP and NADH depletion increase, accompanied by elevated oxidative stress and cell death (Tian et al., 2013).

### 3.4 Angiogenesis

Following MI, angiogenesis is often limited, and significant angiogenic impairment can contribute to systolic dysfunction in the context of heart failure (Yan et al., 2020). The formation of a capillary network is crucial after MI. Impaired neovascularization limits blood supply to damaged myocardium, exacerbating ischemia and promoting adverse remodeling. Factors such as Apelin regulate cardiac angiogenesis, and deficiency in such factors can compromise endothelial sprouting and overall myocardial angiogenesis, contributing to heart failure (Wang et al., 2013a). Research on MSC-Exos has demonstrated their capacity to promote cardioprotection and angiogenesis following MI. These exosomes upregulate VEGF and neovascularization, improving cardiac function and reducing infarct size. (Kang et al., 2015).

**FIGURE 1:**
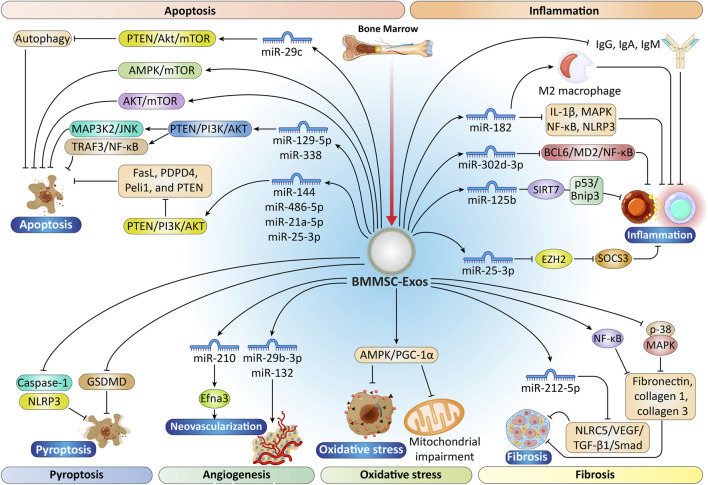
Schematic illustration of the intricate regulatory pathways of BMMSC-Exos in cardiac damage and repair. BMMSC-Exos carry a spectrum of microRNAs and biomolecules involved in controlling apoptosis, autophagy, and pyroptosis *via* pathways such as PTEN/Akt/mTOR, MAP3K2/JNK, PI3K/AKT, and Caspase-1/NLRP3. These exosomes also suppress inflammation by modulating macrophage polarization and suppressing pro-inflammatory cytokines (e.g., IL-1β, TNF-α), and enhance immunomodulation *via* NF-κB, MAPK, SIRT7, SOCS3, and EZH2 targeting. Besides that, BMMSC-Exos facilitate angiogenesis by enhancing neovascularization-related factors (e.g., Efna3, VEGF) and abolish oxidative stress by modulating AMPK/PGC-1α-mediated mitochondrial activity. Fibrosis is controlled through the TGF-β1/Smad and p38 MAPK signaling pathways, resulting in reduced fibronectin and collagen deposition. Collectively, these mechanisms demonstrate the therapeutic potential of BMMSC-Exos to rescue myocardium and regenerate tissue through synergistic molecular mechanisms. BMMSCs, bone marrow-derived MSCs; PTEN, phosphatase and tensin homolog; AKT, protein kinase B; mTOR, mechanistic target of rapamycin kinase; JNK, jun N-terminal kinase; PI3K, phosphoinositide 3-kinase; NLRP3, NLR family pyrin domain containing 3; IL-1β, Interleukin-1 beta; TNF-α, Tumor Necrosis Factor Alpha; NF-κB, nuclear factor kappa-light-chain-enhancer of activated B cells; MAPK, phosphorylated p38 mitogen-activated protein kinase; SOCS3, suppresses inhibitor of cytokine signaling 3; VEGF, vascular endothelial growth factor; AMPK/PGC-1α, adenosine monophosphate-activated protein kinase/peroxisome proliferator-activated receptor-gamma coactivator 1 alpha; TGF-β, Transforming growth factor β.“Created with Adobe Illustrator 27.6.1”.

Given MSC-Exos’ potential significance in injury response, tissue repair, and remodeling, a substantial body of preclinical and clinical studies is investigating their application in tissue regeneration. Next, as illustrated in Figure 1 and Table 1, we elaborate on the role of MSC-Exos with a focus on sources from BMMSCs, ADSCs, and HUCMSCs in MI from several perspectives.

**TABLE 1:** Healing potential of exosomes in cardiac injuries: Mechanisms, doses, and delivery routes.

​	Model of myocardial injury	Dose and route	Molecular mechanism	Related miRNAs	References
BMMSC	Mice	50 µg Intramyocardial injection	Promoting the differentiation of macrophages into the M2 phenotype	miR-21-5p	Shen and He (2021)
	I/R injury model	5 μg, injected into 5 sites at the border of the infract	Targets SIRT7 → suppressing inflammation/apoptosis • Regulates p53/Bnip3 → inhibiting autophagy	miR-125b	Xiao et al. (2018)
	Myocardial I/R injury	50 μg MSC-Exo, intramyocardial injection	Modulates TLR4/NF-κB/PI3K/AKT → promoting M2 polarization	miR-182	Zhao et al. (2019a)
	Ischemic myocardium	5 μg MSC-Exo, administered into the peripheral region of the infarcted heart	Downregulates EZH2 → Suppresses SOCS3	miR-25-3p	Peng et al. (2020a)
	MI model	​	Modulates NLRC5/VEGF/TGF-β1/Smad → ↓TGF-β1 → ↓ECM deposition and fibrosis	miR-212-5p	Wu et al. (2022)
	Acute MI	injected near the infarct area	Regulates BCL6/MD2/NF-κB → modulating inflammation	miR-302d-3p	Liu et al. (2022)
	Hypoxic cardiomyocytes	12.5 μg protein, injected into the pericardial sac. 5 μg in 100 μL PBS, injected into the border zone of the infarcted heart	Downregulates FasL, PDPD4, Peli1, PTEN → ↓apoptosis	miR-21a-5p, miR-25-3p	Luther et al. (2018), Peng et al. (2020b)
	MI (Rats)	50 μL, myocardium	regulate TRAF3/NF-κB and MAP3K2/JNK → inhibit apoptosis	miR-129-5p, miR-338	Yan et al. (2022), Fu et al. (2020)
	H/R injury	20 μg, administered on both sides of the border zones immediately following LAD coronary artery ligation	Regulates PTEN-Akt-mTOR → controls autophagy	miR-29c	Li et al. (2020)
	I/R injury and H/R cells	10 μg, intramyocardially	Inhibits MEKK1-MKK4-JNK → ↓autophagy-related death	miR-455-3p	Wang and Shen (2022)
	MI model	100 μL, tail intravenous injection	Regulates miR-138-5p/SIRT1 → ↓ inflammatory responses, and pyroptosis	lncRNA KLF3-AS1	Mao et al. (2019)
	H/R-induced MI	​	Modulates miR-556-5p/XIAP → ↓H/R injury	lncRNA A2M-AS1	Yu et al. (2023)
	MI	Single IV injection	miR-210-Efna3 pathway →promotes neovascularization	miR-210	Wang et al. (2017b)
	Ischemic cardiac tissue	600 μg Transplantation into tissue	Targets RASAL1 → stimulates new blood vessel formation	miR-132 (mimics)	Ma et al. (2018)
	MI (Rats)	​	Targets ADAMTS16 → angiogenesis	miR-29b-3p	Zheng et al. (2022a)
	Rat acute MI model	80 μg, Intramyocardial injection	Improved blood flow; ↓ Infarct size	​	Bian et al. (2014)
ADSCs	Myocardial injury	400 *μ*g, tail intravenous injection	Downregulates inflammatory factors	miR-126	Luo et al. (2017)
	MI	100 μg, administered in the border region of the infarcted heart close to the ligation site	• Targets TGFBR2/Smad2 pathway • Prevents Smad2/3 phosphorylation • Disrupts TGF-β	miR-671	Wang et al. (2021c)
	MI site	​	Suppresses Bcl2L11 (pro-apoptotic) and SLC8a1 (calcium overload)	miR-214	Shukla et al. (2017)
	I/R injury	2.8 × 10^9^ and 1.56 × 10^9^ exosomes, midclavicular line	Suppresses EGR1 → Inhibits apoptosis	miR-146a	Xiao et al. (2016)
	Hindlimb ischemia (HLI) model	5 μg, 2.2 × 10^7^ particles., intramyocardially injection, 100 μg, Intravenous injection	• Activates FIH1/HIF-1α pathway • Promotes endothelial migration/tube formation	miR-31	Zhu et al. (2022)
	Matrigel plug assay	100 μg/mL, subcutaneously in the dorsal region of female nude mice	• Inhibits DLL4 (angiogenesis inhibitor) • Promotes endothelial tip cell formation	miR-125a	Liang et al. (2016)
HUCMSCs	Rat I/R injury	​	Downregulates KEAP1/Nrf2/HO-1 pathway → Promotes M2 polarization and cardioprotection	miR-24	Hou et al. (2024)
	Mouse myocardial I/R injury	200 μg, intramyocardial injection	Targets c-Fos → Inhibits the inflammatory response and increases Treg proportion	miR-181a	Wei et al. (2019)
	AMI model	5ug, delivered into the peripheral region of the infarcted heart	Suppresses NLRP3 inflammasome *via* FOXO3 downregulation → Inhibits pyroptosis	miR-100-5p	Song et al. (2021)
	AMI model	400 μg, injected *via* the tail vein	Upregulates Smad7 by inhibiting miR-125b-5p → Enhances myocardial regeneration	miR-125b-5p	Wang et al. (2018)
	AMI tissue	400 μg/g	Targets SOX6 → Activates AKT and inhibits JNK3/caspase-3 → Reduces apoptosis	miR-19a	Huang et al. (2020)
	Hypoxia-induced apoptosis	400 μg, tail vein	Target PTEN → Activate PI3K/AKT pathway → Inhibit hypoxia-induced cardiomyocyte apoptosis	miR-144 miR-486-5p	Wen et al. (2020), Sun et al. (2019)
	​	​	Enhances cell viability and reduces apoptosis	miR-24	Bhaskara et al. (2023)
	Permanent occlusion of the left anterior descending (LAD) coronary artery	20 μg, administered into two sites within the border region of the infarcted myocardium	Targets PRSS23/Snail/α-SMA axis → Promotes angiogenesis	miR-1246	Wang et al. (2021d)

Other sources					
Human amniotic fluid MSC-Exos (hAFMSC-Exos)	Isoproterenol-induced cardiac fibrosis (rat)	100 μg, *via* the tail vein	↑ HIF-1α/VEGF expression → Enhanced angiogenesis	​	Hu et al. (2021)
Human placental MSC-Exos (hPMSC-Exos)	MI	200 µL of hPMSC-Exos *via* tail vein injection (administered on days 7, 9, and 11 post-MI)	↓ Myocardial fibrosis/LV remodeling; ↓ AST/BNP/MYO/Tn-I/TC; ↓ IL-1β/IL-6/TNF-α/MCP-1; ↑ HDL	​	Yang et al. (2022b)
Human Endometrial MSC-Exos (hEnMSC-Exos)	MI	1 × 10^6^ cells *via* intramyocardial injection	↑ miR-21 delivery → ↓ PTEN/↑ p-Akt in CMs and ECs; ↓ apoptosis (PTEN/Akt pathway); ↑ angiogenesis	miR-21	Wang et al. (2017a)
	MI	80 μg, intramyocardial injection	Inhibits H_2_O_2_-induced H9c2 apoptosis; ↑ HUVEC angiogenesis; EGF/PI3K/AKT pathway activation	miR-21	Yan et al. (2024a)
	MI	300 μg, Local intramyocardial injection (3 injection sites around the infarct)	↓ Fibrosis size; ↑ histological scores; prevents cardiac remodeling	​	Sepehri et al. (2025)

Abbreviations: TLR4, toll-like receptor 4; NF-κB, nuclear factor kappa-light-chain-enhancer of activated B cells; PI3K, phosphoinositide 3-kinase; AKT, protein kinase B; SOCS3, suppresses inhibitor of cytokine signaling 3; VEGF, vascular endothelial growth factor; TGF-β, Transforming growth factor β; BCL6, B-cell lymphoma 6 protein; MD2, myeloid differentiation protein 2; PDPD4, protein disulfide isomerase family A member 4; Peli1, pellino E3 ubiquitin protein ligase 1; PTEN, phosphatase and tensin homolog; TRAF3, TNF receptor-associated factor 3; MEKK1, mitogen-activated protein kinase kinase kinase 1; EGR1, early growth response factor 1; FIH1, factor inhibiting; HIF-1 alpha, hypoxia-inducible factor 1-alpha; KEAP1, kelch-like ECH-associated protein 1; Nrf2/HO-1, nuclear factor erythroid 2-related factor 2/heme oxygenase-1 pathway; NLRP3, NLR family pyrin domain containing 3; PRSS23/Snail/α-SMA, serine protease 23/snail family transcriptional repressor/alpha-smooth muscle actin. HIF-1 alpha, hypoxia-inducible factor 1-alpha; VEGF, vascular endothelial growth factor; IL-1β, Interleukin-1 beta; TNF-α, Tumor Necrosis Factor Alpha; HDL, high-density lipoprotein; PTEN, phosphatase and tensin homolog; hAFMSC-Exos, Human amniotic fluid MSC-Exos; hPMSC-Exos, Human placental MSC-Exos; hEnMSC-Exos, Human Endometrial MSC-Exos.

### 3.5 BMMSC-derived exosomes

The anti-inflammatory, antioxidant, anti-apoptotic, anti-fibrotic, and pro-angiogenic properties of exosomes motivated researchers to examine how BMMSC-Exos influence cardiac function. In this section, we discuss the results and provide a concise summary in Figure 1 and Table 1.

#### 3.5.1 Anti-inflammation

BMMSC-Exos also significantly inhibit the proliferation of CD3^+^ T cells. The observed effect may result from upregulation of the cyclin-dependent kinase inhibitor p27kip1and downregulation of cyclin-dependent kinase 2 (CDK2), contributing to cell-cycle arrest in T lymphocytes (Lee et al., 2020). Comparable results have been observed in prior *in vitro* studies examining the interaction between BMMSC-Exos and peripheral blood mononuclear cells, which observed that MSCs caused apoptosis in CD3^+^ T cells, while also suppressing CpG-stimulated B cell proliferation, differentiation, and immunoglobulin secretion (IgG, IgA, IgM) (Budoni et al., 2013). Moreover, several studies indicate that BMMSC exosome therapy markedly reduces levels of pro-inflammatory mediators such as Interleukin-1 beta (IL-1β), nuclear factor kappa-light-chain-enhancer of activated B cells (NF-κB), phosphorylated p38 mitogen-activated protein kinase (MAPK), and components of the NLR family pyrin domain containing 3 (NLRP3) inflammasome (Kore et al., 2021). Additionally, investigations have shown that delivering BMMSC-Exos directly into the infarcted myocardium in mice reduces inflammatory responses by promoting macrophage polarization toward the anti-inflammatory M2 phenotype through the transfer of miR-21-5p, thereby supporting cardiac repair processes (Shen and He, 2021). In this context, exosomes upregulating fibronectin type III domain-containing protein five exert additional anti-inflammatory effects by inhibiting NF-κB signaling and activating the nuclear factor erythroid 2-related factor 2/heme oxygenase-1 (Nrf2/HO-1) pathway, thereby promoting M2 macrophage polarization and contributing to myocardium healing (Ning et al., 2021). BMMSC-Exos that carry miR-125b have been shown to restore cardiac function in I/R injury models by suppressing inflammation and apoptosis in cardiomyocytes, primarily by focusing on SIRT7 (Xiao et al., 2018). Additionally, research has demonstrated that miR-182 contained within BMMSC-Exos modulates the toll-like receptor 4 (TLR4)/NF-κB/phosphoinositide 3-kinase (PI3K)/protein kinase B (AKT) signaling pathway, leading to reduced myocardial I/R damage through the promoting the polarization of inflammatory macrophages toward the anti-inflammatory M2 phenotype (Zhao et al., 2019a).

In addition, miR-125b, found within BMMSC-Exos, is instrumental in inhibiting excessive autophagy by regulating the phosphorylated p53/B-cell lymphoma 2 (BCL2)-interacting protein three (p53/Bnip3) signaling pathways. This regulatory mechanism plays a crucial role in dampening the inflammatory response following myocardial I/R injury (Xiao et al., 2018). Additionally, miR-25-3p within BMMSC-Exos suppresses inhibitor of cytokine signaling 3 (SOCS3) expression by inhibiting enhancer of zeste homolog 2 (EZH2), thereby further attenuating inflammation in the ischemic myocardium (Peng et al., 2020a). MicroRNA-302d-3p within BMMSC-Exos participates in modulating the inflammatory microenvironment post-acute MI through the regulation of the B-cell lymphoma 6 protein (BCL6)/myeloid differentiation protein 2 (MD2)/NF-κB signaling axis, ultimately mitigating adverse ventricular remodeling (Liu et al., 2022).

#### 3.5.2 Anti-fibrosis

Transforming growth factor β (TGF-β) molecules are multifunctional cytokines that play a significant role in tissue fibrosis (Dobaczewski et al., 2011). TGF-β1, in particular, is crucial during various Phases of wound healing and scar development (Li et al., 2016). As shown in Figure 1, Smad2 and Smad3 act as the principal downstream mediators of TGF-β1, critically regulating collagen expression and driving tissue fibrosis (Meng et al., 2010; Hu et al., 2020). MiR-212-5p derived from BMMSC-Exos alleviates fibrosis induced by MI through the modulation of the NLR family CARD domain containing 5 (NLRC5)/VEGF/TGF-β1/Smad axis. Decreases in TGF-β1 directly correlate with reductions in ECM deposition and fibrosis (Wu et al., 2022). BMMSC-Exos can significantly diminish interstitial and perivascular fibrosis in the ischemic myocardium, along with a decrease in fibronectin expression within both infarcted and peri-infarct regions. The mechanisms responsible for these effects involve the downregulation of p-38 MAPK and NF-κB activation, resulting in suppression of ECM components such as fibronectin, collagen 1, and collagen 3 (Kore et al., 2021).

#### 3.5.3 Anti-oxidative stress

Gene Ontology (GO) and Kyoto Encyclopedia of Genes and Genomes (KEGG) enrichment analyses revealed that BMSCs-Exos may alleviate I/R injury in the myocardium by activating the adenosine monophosphate-activated protein kinase/peroxisome proliferator-activated receptor-gamma coactivator 1 alpha (AMPK/PGC-1α) signaling pathway. The enrichment results, supported by both *in vitro* and *in vivo* assays, are summarized in Figure 1, which illustrates the involvement of this pathway in regulating mitochondrial function and cardioprotection. Compound C is an AMPK suppressor, and sh-AMPK (a short hairpin RNA that silences AMPK) both inhibit the activation of PGC-1α and its downstream targets, thereby nullifying the protective effects of exosomes against oxidative stress and mitochondrial dysfunction in injured cardiomyocytes. To demonstrate that the protective effects of BMSCs-Exos depend on activation of this pathway, researchers inhibited AMPK using Compound C and sh-AMPK and showed that pathway activation is crucial for the protective effects of BMSCs-Exos, which are AMPK-dependent; silencing AMPK did not alter the expression of its phosphorylated form but did indicate a lack of protective effect when AMPK was nonfunctional. Conversely, silencing PGC-1α did not influence the expression of phosphorylated AMPK (p-AMPK). BMSCs-Exo can decrease oxidative stress and mitochondrial impairment in cardiomyocytes by stimulating the AMPK/PGC-1α signaling pathway, ultimately protecting against myocardial I/R injury (Zhuang et al., 2025; Jiang et al., 2025).

#### 3.5.4 Anti-apoptotic

The PI3K/AKT pathway plays a crucial role in regulating apoptosis and cell viability. Additionally, miR-144 and miR-486-5p within BMMSC-Exos significantly inhibit hypoxia-induced apoptosis in cardiomyocytes *via* the phosphatase and tensin homolog (PTEN)/PI3K/AKT pathway (Sun et al., 2019; Wen et al., 2020) The mechanistic involvement of these microRNAs in modulating PTEN activity and enhancing cardiomyocyte survival is summarized in Figure 1. MiR-21a-5p and miR-25-3p, found within BMMSC-Exos, have been shown to reduce apoptosis in hypoxic cardiomyocytes. This effect is attained through downregulation of pro-apoptotic gene expression, specifically targeting genes such as Fas ligand (FasL), protein disulfide isomerase family A member 4 (PDPD4), pellino E3 ubiquitin protein ligase 1 (Peli1), and PTEN (Luther et al., 2018; Peng et al., 2020b). Moreover, miR-129-5p and miR-338 are notably enhanced in BMMSC-Exos and suppress myocardial apoptosis while enhancing cardiac function in MI rats through regulation of the TNF receptor-associated factor 3 (TRAF3)/NF-κB and MAP3K2/c-jun N-terminal kinase (JNK) signaling pathways, respectively (Yan et al., 2022; Fu et al., 2020).

Recent studies indicate that autophagy is crucial for preserving the structure and function of cardiomyocytes, with enhanced autophagy offering protective benefits to the heart during episodes of MI and hypoxia (Wu et al., 2019). BMMSC-Exos have been shown to modulate the cardiac microenvironment post-MI by enhancing autophagy in injured cardiac tissues. This effect appears to correlate with increased expression of autophagy-related protein 13 in H9c2 cells following MI induction (Zou et al., 2019). BMMSC-Exos contribute to the reduction of apoptosis in cardiomyocytes by activating the AMPK/mechanistic target of rapamycin kinase (mTOR) and AKT/mTOR signaling pathways, thereby inducing autophagy. It is noteworthy that normal levels of autophagy are crucial for protecting the heart; however, excessive autophagy can lead to cell death and drive ventricular remodeling (Fang et al., 2022; Liu et al., 2017). BMMSC-Exos exhibit elevated levels of miR-29c, with expression changes observed after cell treatment under hypoxic/reoxygenation (H/R) conditions. Furthermore, *in vivo* I/R studies validated changes in miR-29c expression and the activation of the PTEN-Akt-mTOR pathway, a key regulator of autophagic alterations during these processes (Li et al., 2020).

Pyroptosis is also a type of PCD characterized by the release of inflammatory cytokines, notably caspase-1 and NLR family pyrin domain containing 3 (NLRP3) (Audia et al., 2018; Tang et al., 2020). Research has shown that human BMMSC-Exos significantly decrease the expression of proteins associated with pyroptosis, including caspase-1 and NLRP3, thereby providing protective effects against I/R injury in the myocardium (Tang et al., 2020). These findings are summarized in Figure 1. In particular, miR-125b has been shown to inhibit the expression of pro-apoptotic factors such as p53 and BCL2 antagonist/killer 1 (BAK1), thereby reducing cardiomyocyte apoptosis and improving cardiac function post-I/R injury (Zhang et al., 2021a).

Overexpression of mitogen-activated protein kinase kinase kinase 1 (MEKK1) significantly decreased cell apoptosis, diminished cellular survival, inhibited autophagy activation, and reduced the protective effects of exosomal miR-455-3p on H/R myocardial cells. Upregulation of miR-455-3p inhibited the MEKK1-mitogen-activated protein kinase kinase 4 (MKK4)-JNK signaling pathway. The expression of miR-455-3p was found to be downregulated in exosomes derived from BMMSCs, as well as in I/R myocardial tissues and H/R myocardial cells. Enrichment of miR-455-3p in BMMSC-Exos was shown to reduce cardiomyocyte damage and autophagy-related cell death induced by H/R conditions. *In vivo* studies also demonstrated that BMMSC-Exos enriched with miR-455-3p attenuated myocardial injury and improved myocardial cell function following I/R injury (Wang and Shen, 2022).

BMMSC-Exos mitigate ischemic myocardial damage and pyroptosis by specifically targeting gasdermin D (GSDMD) and lowering its expression levels (Bhaskara et al., 2023). Additionally, the upregulation of divalent metal transporter 1 (DMT1) has been linked to the promotion of ferroptosis induced by H/R (Wang et al., 2024). Certain lncRNAs have been implicated in the cardioprotective effects conferred by exosomes. Notably, the lncRNA KLF3 antisense RNA 1 (KLF3-AS1) found in hMSC-Exos has the capacity to inhibit cell viability, inflammatory responses, and pyroptosis in cardiomyocytes. This is achieved *via* the regulation of the miR-138-5p/sirtuin 1(SIRT1) axis, which ultimately helps to inhibit the progression of MI (Mao et al., 2019). BMMSC-Exos containing lncRNA alpha-2-macroglobulin antisense RNA 1 (A2M-AS1) have been shown to alleviate H/R-induced MI through the modulation of the miR-556-5p/X-linked inhibitor of apoptosis (XIAP) axis (Yu et al., 2023). Furthermore, previously stated mechanisms of cell death, cuproptosis, represent a new form of cell death that is intricately linked to mitochondrial respiration (Yuan et al., 2022). The potential connection between exosomes and mechanisms in inhibiting cuproptosis has not yet been established and can be reviewed and for future research into the functions of exosomes in cellular dynamics.

#### 3.5.5 Pro-angiogenic

Given that myocardial angiogenesis following MI is restricted, substantial impairment in angiogenesis can lead to systolic dysfunction following heart failure (Yan et al., 2020).

In a Sprague-Dawley rat model of induced acute MI (AMI), it was observed that BMMSC-Exos treated conditions significantly increased the density of newly formed functional capillaries. Additionally, this intervention suppressed cell proliferation, thereby modulating T-cell activity, reducing apoptosis, and ultimately facilitating the recovery of blood flow (Teng et al., 2015). In a rat model of MI, BMMSC-Exos protected cardiomyocytes from ischemic injury, both *in vitro* and *in vivo*. This protective mechanism was linked to their effects on cardiac tissue and vasculature, promoting cardiac regeneration through neovascularization and anti-vascular remodeling, as well as reducing reperfusion injury (Kang et al., 2015; Farahzadi et al., 2024). In a porcine MI model, intramyocardial delivery of BMMSC-Exos led to higher capillary density and improved blood flow to the ischemic region of the myocardium. These effects were mediated through the upregulation of the MAPK and AKT/endothelial nitric oxide synthase (eNOS) signaling pathways, ultimately leading to an increase in cardiac output (Potz et al., 2018). Also, in a Yorkshire pig model of chronic myocardial ischemia and metabolic syndrome, intramyocardial injection of human BMMSC-Exos raised vascular density, improved blood flow, and enhanced cardiac function in the affected cardiac regions (Scrimgeour et al., 2019). A one-time intravenous (IV) dose of BMMSC-Exos can enhance cardiac function and angiogenesis in mice with MI, likely through the miR-210-Efna3 pathway that supports neovascularization in the infarcted heart (Wang et al., 2017b). Furthermore, in ischemic myocardial tissue, BMMSC-Exos carrying miR-132 mimics substantially promote neovascularization around the infarct zone *via* modulation of RASAL1 gene expression (Ma et al., 2018). Higher levels of miR-29b-3p in BMMSC-Exos also boost angiogenesis and influence ventricular remodeling in rat MI models, through targeting ADAMTS16, a metalloproteinase involved in ECM remodeling (Zheng et al., 2022a). All of the aforementioned effects of BMMSC-Exos on cardiac function and angiogenesis are illustrated in Figure 1. Notwithstanding, several studies report that intravenous BMMSC-Exos delivery fails to increase microvascular density in the ischemic myocardium despite upregulation of multiple pro-angiogenic signaling pathways. This discrepancy may arise from differences between systemic *versus* direct intramyocardial administration, as intravenous routes may differentially affect angiogenic signaling (Scrimgeour et al., 2020). Additional evidence from rat models of myocardial I/R and *in vitro* models of H/R in myocardial microvascular endothelial cells indicates that BMMSC-Exosomes promote microvascular repair during stress by modulating platelet-derived growth factor receptor beta (PDGFR-β), thereby reducing fibrosis after I/R and improving cardiac performance. Importantly, the early stimulation of the vascular growth factor pathway, especially through PDGF-BB/PDGFR-β, can foster regeneration of functional tissues, yet excessive stimulation risks fibrotic remodeling (Wang et al., 2021b).

### 3.6 ADSCs-derived exosomes

**FIGURE 2:**
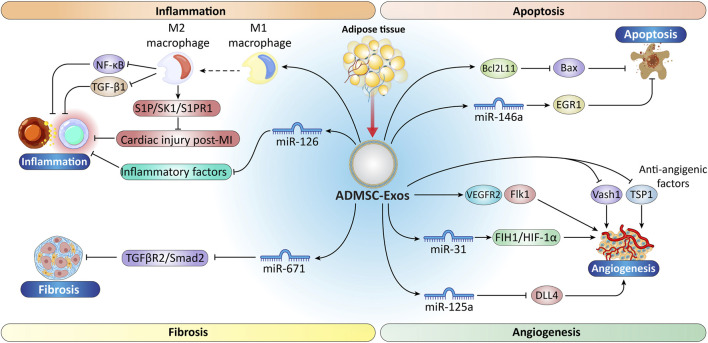
Schematic representation of the molecular processes by which ADMSC-Exos control cardiac injury and repair after MI. ADMSC-Exos modulate inflammation by promoting M2 macrophage polarization and suppressing inflammatory signaling *via* mechanisms such as NF-κB, TGF-β1, and S1P/SK1/S1PR1, thereby reducing myocardial injury. In apoptosis, ADMSC-Exos regulate gene expression (Bcl2L11, EGR1, Bax) and deliver miR-212-5p to suppress cell death. The exosomes also attenuate fibrosis by inhibiting TGF-β1/TGFβR2/Smad2 signaling *via* miR-212-5p. Additionally, ADMSC-Exos promote angiogenesis by enhancing VEGFR2/Flk1 and DLL4 networks and by modulating miR-31, miR-125a, FIH1/HIF-1α, Vash1, and TSP1. Collectively, these actions illustrate the therapeutic potential of ADMSC-Exos in reducing inflammation and apoptosis, inhibiting fibrosis, and promoting vascular remodeling after cardiac injury. ADMSCs, adipose-derived mesenchymal stem cells; NF-κB, nuclear factor kappa-light-chain-enhancer of activated B cells; TGF-β, Transforming growth factor β; S1P/SK1/S1PR1, sphingosine-1-phosphate/sphingosine kinase 1/sphingosine-1-phosphate receptor 1; FIH1, factor inhibiting; HIF-1 alpha, hypoxia-inducible factor 1-alpha; Vash1, vasohibin 1; TSP1, thrombospondin 1. “Created with Adobe Illustrator 27.6.1”.

The availability of BMMSCs and their application in research and clinical settings are limited due to the invasive harvesting procedure, which requires general anesthesia (Ullah et al., 2015). In contrast, ADSCs demonstrate comparable differentiation potential to BMMSCs, being capable of differentiating into ectodermal, mesodermal, and endodermal lineages, and also exhibit notable immunomodulatory properties (Li et al., 2015). The mechanisms of the effect of ADSC-Exos on heart tissue repair and the studies conducted in this field are shown in Figure 2 and Table 1.

#### 3.6.1 Anti-inflammation

The immunomodulatory effects of ADSC-Exos may be linked to macrophage polarization. ADSC-Exos efficiently induce the shift of macrophages to the M2 phenotype, thereby suppressing inflammatory responses and mitigating myocardial fibrosis through downregulation of NF-κB and TGF-β1 expression (Deng et al., 2019). ADSC-Exos stimulated with IFN-γ and TNF-α exhibit increased immunosuppressive and anti-inflammatory effects (Domenis et al., 2018). Furthermore, ADSC-Exos overexpressing miR-126 have been shown to downregulate inflammatory factors and alleviate myocardial injury (Luo et al., 2017). The protective role of ADSC-Exos in improving cardiac injury post-MI, mediated through activation of the S1P/SK1/S1PR1 signaling pathway and enhanced macrophage polarization toward the M2 phenotype, is summarized in Figure 2, which illustrates their capacity to suppress inflammation and promote myocardial repair (Deng et al., 2019).

#### 3.6.2 Anti-fibrosis

TGF-β is regarded as a central mediator of fibrotic processes. As depicted in Figure 2, TGF-β triggers signaling cascades that activate the Smad family proteins, which govern the transcription of genes responsible for extracellular matrix (ECM) production. Elevated TGF-β concentrations consistently associate with greater ECM deposition, a hallmark of fibrotic states (Xu et al., 2016). Among Smad proteins, Smad3 is particularly important in promoting fibrogenesis. It is activated by TGF-β and promotes the expression of fibrosis-associated genes, including those encoding collagen. Conversely, Smad2 has a protective role against fibrosis, as its deletion enhances TGF-β/Smad3 signaling and increases collagen expression (Meng et al., 2010). Decapentaplegic homolog 7 (Smad7) acts as an inhibitory Smad, counteracting the effects of Smad3 and thus playing a protective role in fibrosis (Fukasawa et al., 2004). Modulating the TGF-β/Smad signaling pathway offers a promising therapeutic approach for fibrosis management. MiR-671, encapsulated in ADMSC-Exos, directly inhibits the TGFβR2/Smad2 pathway, reducing myocardial fibrosis in mice with MI and mitigating ischemic heart damage. This effect arises from inhibiting the Smad2/3 phosphorylation by TGF-β1, from disrupts the interactions between TGF-β receptors and additional Smad proteins (Wang et al., 2021c).

#### 3.6.3 Anti-apoptotic

Research indicates that ADMSC-Exos can modulate the sphingosine-1-phosphate/sphingosine kinase 1/sphingosine-1-phosphate receptor 1 (S1P/SK1/S1PR1) signaling pathway. Significantly, miR-214, which shows elevated expression in these exosomes, can suppress the expression of BCL2-like 11 (apoptosis facilitator, BCL2L11) and solute carrier family 8 member A1 (SLC8a1) upon delivery to the MI site. The protein encoded by the BCL2L11 gene is known to induce apoptosis through Bax activation or by counteracting anti-apoptotic proteins, thereby playing a key role in regulating cell death. (Shukla et al., 2017). During cardiac stress, the sodium/calcium exchanger protein encoded by the solute carrier family 8 member A1 (SLC8A1), facilitates cardiomyocyte death linked to excessive calcium accumulation. Studies using ADMSC-Exos have demonstrated their ability to inhibit apoptosis in hypoxic cardiomyocytes by delivering overexpressed miR-146a. The primary mechanism underlying the anti-apoptotic effect of miR-146a in I/R-injured tissue is its inhibition of early growth response factor 1 (EGR1) expression (Xiao et al., 2016).

#### 3.6.4 Pro-angiogenic

A study has shown that treating of human umbilical vein endothelial cells (HUVECs) with ADMSC-Exos enhances the expression of proangiogenic genes angiopoietin 1 (Ang1) and fetal liver kinase 1 (VEGF Receptor 2, Flk1), while antiangiogenic genes vasohibin 1 (Vash1) and thrombospondin 1 (TSP1) are downregulated (Luo et al., 2017; Zhong et al., 2024). On the other hand, miR-31 activates the factor inhibiting HIF-1 alpha (FIH1)/HIF-1α signaling pathway, which is critical for angiogenic processes. This activation enhances the transcriptional activity of HIF-1α, stimulating endothelial cell migration and vascular tube formation. Together, these microRNAs promote a pro-angiogenic environment, enhancing the capacity of endothelial cells to form new blood vessels, which is vital for tissue repair and regeneration (Zhu et al., 2022). In particular, miR-125a, enriched in ADMSC-derived exosomes, suppresses the expression of the angiogenesis inhibitor delta-like 4 (DLL4) by targeting its 3′UTR, thereby enhancing angiogenesis by promoting endothelial tip cell formation (Liang et al., 2016). These findings are highlighted in Figure 2, which illustrates how ADMSC-Exos and their microRNA cargo orchestrate multiple signaling pathways to stimulate angiogenesis and support myocardial repair.

### 3.7 HUCMSCs-derived exosomes

**FIGURE 3:**
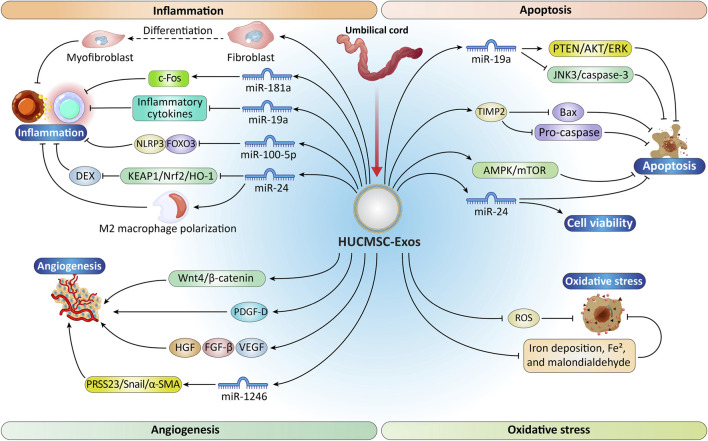
Schematic representation of the molecular processes by which HUCMSC-Exos modulate cardiac injury and repair after MI. HUCMSC-Exos trigger anti-inflammatory effects by promoting M2 macrophage polarization and suppression of pro-inflammatory cytokines by miRNAs such as miR-181a, miR-19a, miR-100-5p, and miR-24, thereby influencing fibroblast differentiation and suppressing inflammasome activation (NLRP3). They also promote cell survival by regulating apoptosis-related signaling pathways including PTEN/AKT/ERK, JNK3/caspase-3, Bax, TIMP2, and AMPK/mTOR through miR-19a and miR-24 activities. Oxidative stress is attenuated by reducing ROS and inhibiting iron deposition and lipid peroxidation markers such as malondialdehyde. Furthermore, HUCMSC-Exos stimulate angiogenesis by delivering pro-angiogenic factors and miRNAs including VEGF, FGF-β, HGF, PDGF-D, Wnt4/β-catenin, and miR-1246, regulating targets such as PRSS23, Snail, and α-SMA to promote vascular regeneration, collectively supporting inflammation resolution, apoptosis inhibition, oxidative-stress reduction, and angiogenesis enhancement for tissue repair. HUCMSCs, human umbilical cord MSCs; PTEN, phosphatase and tensin homolog; AKT, protein kinase B; ERK, The extracellular signal-regulated kinase; AMPK, adenosine monophosphate-activated protein kinase; VEGF, vascular endothelial growth factor; FGF-β, basic fibroblast growth factor; HGF, hepatocyte growth factor; PDGF-D, platelet-derived growth factor-D; PRSS23/Snail/α-SMA, serine protease 23/snail family transcriptional repressor/alpha-smooth muscle actin. “Created with Adobe Illustrator 27.6.1”.

In comparison with other mesenchymal stem cells, hUCMSC-derived exosomes offer advantages such as cost-effectiveness, minimal invasiveness, straightforward isolation, high cellular yield, efficient gene transfection, and low immunogenicity, generating considerable interest among researchers for tissue repair applications (Hoffmann et al., 2017). The mechanisms of the effect of hUCMSC-Exos on heart tissue repair and the studies conducted in this field are shown in Figure 3 and Table 1.

#### 3.7.1 Anti-inflammation

During the inflammatory phase following MI, hUCMSC-Exos also promote fibroblast differentiation into myofibroblasts within the infarcted region, thereby decreasing the inflammatory response of cardiomyocytes and aiding tissue repair (Shi et al., 2019).

As shown in Figure 3, exosomes derived from hUCMSCs represent a highly promising therapeutic avenue for acute myocardial infarction (AMI). MiR-19a, which inhibits myocardial cell apoptosis, is found at lower levels in AMI-affected myocardial tissues compared with healthy tissues. However, hUCMSC-Exos markedly enhance miR-19a release, mitigating ischemic damage and reducing the expression of inflammatory cytokines (Huang et al., 2020). Due to the limited experimental data currently available, hUCMSC-Exos exhibit robust immunoregulatory properties, making them highly promising for AMI treatment and warranting further investigation (Xiong et al., 2021).

Molecularly, miR-24 suppresses the expression of kelch-like ECH-associated protein 1 (KEAP1) by direct targeting, which disrupts the Nrf2/HO-1 signaling cascade through KEAP1 downregulation. In rat models of myocardial I/R injury, *in vivo* experiments showed that hUCMSC-Exos delivering miR-24 significantly potentiated dexamethasone (DEX) preconditioning’s anti-inflammatory and anti-apoptotic actions. Collectively, these results indicate that miR-24, when transported *via* hUCMSC-Exos, promotes M2 macrophage polarization and enhances DEX-mediated cardioprotection against I/R injury by inhibiting the KEAP1/Nrf2/HO-1 signaling axis (Hou et al., 2024). These mechanistic insights are summarized in Figure 3, which illustrates how miR-24-enriched hUCMSC-Exos modulate signaling pathways to suppress inflammation, reduce apoptosis, and improve myocardial repair.

As highlighted in Figure 3, these findings demonstrate that hUCMSC-Exos actively modulate the cardiac immune microenvironment following AMI, underscoring their role in post-infarction immunoregulation. Given the established link between miR-181a and inflammatory-related diseases (Ghorbani et al., 2017), miR-181a overexpressing hUCMSC-Exos were used to reduce cardiac injury after I/R. The results showed that Exosome administration promoted an anti-inflammatory milieu, driving the transition from the inflammatory phase to the reparative phase and accelerating wound resolution through M2 macrophage polarization (Kino et al., 2020). In a mouse model of myocardial I/R injury, hUCMSC-Exos engineered to overexpress miR-181a significantly attenuated inflammatory cytokine production and increased cardiac Treg cell frequency *via* direct suppression of the AP-1 transcription factor c-Fos (Wei et al., 2019). Furthermore, hUCMSC-Exos deliver miR-100-5p to suppress cardiomyocyte pyroptosis through FOXO3-mediated transcriptional repression of the NLRP3 inflammasome, culminating in reduced IL-1β and IL-18 release (Song et al., 2021; Liang et al., 2021).

#### 3.7.2 Anti-fibrosis

HUCMSC-Exos have garnered attention for their cardioprotective effects, particularly in AMI and cardiomyocyte hypoxia injury models. One study showed reparative effects of HUCMSC-Exos on myocardial injury by modulating Smad7 expression. As presented in Figure 3, evidence indicated that Smad7 expression is downregulated in the murine infarct border zone and in hypoxia-exposed H9c2(2-1) cardiomyoblasts, confirming its role in post-infarction TGF-β/Smad signaling dysregulation. Concurrently, expression of miR-125b-5p is markedly elevated, a change reversed by hUCMSC-Exos. Moreover, increased cell injury in AMI and hypoxia groups compared with control groups is mitigated by hUCMSC-Exos. By alleviating miR-125b-5p–mediated suppression of Smad7, hUCMSC-Exos restore TGF-β/Smad signaling homeostasis and improve left ventricular function after AMI (Wang et al., 2018).

#### 3.7.3 Anti-apoptotic

An empirical study showed that hUCMSC-Exosomes significantly mitigate the effects of AMI and suppress cardiomyocytes apoptosis. miR-19a is downregulated in AMI tissues and cells, and its level rises following hUCMSC-Exosome treatment. As illustrated in Figure 3, silencing miR-19a within hUCMSC-Exos diminished their protective efficacy against AMI-induced damage. Moreover, SOX-6 was identified as a target of miR-19a, and its suppression alleviated hypoxic injury in H9C2 cardiomyocytes. Co-silencing of both SOX-6 and miR-19a in hUCMSC-Exos activated the AKT pathway while inhibiting the JNK3/caspase-3 signaling axis. In summary, these findings imply that hUCMSC-Exos confer cardiomyocyte protection against AMI-induced injury through miR-19a transfer, SOX-6 targeting, AKT activation, and inhibition of JNK3/caspase-3, offering new insights for AMI therapy (Huang et al., 2020). Additionally, hUCMSC-Exos with enhanced TIMP2 expression significantly boost hypoxic cardiomyocytes resistance to apoptosis by reducing pro-apoptotic proteins Bax and pro-caspase (Xiao et al., 2016). As illustrated in Figure 3, these protective mechanisms converge to enhance cardiomyocyte survival under hypoxic stress, highlighting the therapeutic potential of hUCMSC-Exos in myocardial repair.

HUCMSC-Exos have been shown to mitigate coxsackievirus B3-induced myocarditis by stimulating the AMPK/mTOR signaling pathway, thereby promoting autophagic flux and decreasing cardiomyocyte apoptosis (Gu et al., 2020). hUCMSC-Exos deliver miR-24 to cardiomyocytes, improving cell survival and reducing apoptosis (Wang and Qian, 2014). Decreased expression levels of reactive oxygen species (ROS), iron deposition, Fe^2+^, and malondialdehyde levels have been observed in cardiomyocytes subjected to H/R injury after targeting DMT1 with HUCMSC-Exos (Song et al., 2021). As depicted in Figure 3, these protective mechanisms collectively highlight the multifaceted role of hUCMSC-Exos in reducing oxidative stress, enhancing autophagy, and improving cardiomyocyte viability under pathological conditions.

Research indicates that elevated GATA-4 expression enhances MSC differentiation into cardiac lineages and improves MSC survival in ischemic conditions (Li et al., 2011; Li et al., 2010; Yu et al., 2013a). MSCs with enhanced GATA-4 expression (MSCGATA-4) promote cardiomyocyte survival and decrease apoptosis in the ischemic myocardium (Yu et al., 2013b). Exosomes isolated and purified from human umbilical cord-derived MSCs engineered to express GATA-4 (ExoGATA-4) and from control MSCs (ExoNull) were examined for their effects on cellular injury in neonatal rat cardiomyocytes well as in the rat heart. ExoGATA-4 significantly improved cardiomyocyte viability, reduced apoptosis, and maintained mitochondrial membrane potential under hypoxic conditions. Notably, several anti-apoptotic miRNAs were markedly upregulated in ExoGATA-4, with miR-19a notably elevated, in cardiomyocytes and myocardium treated with ExoGATA-4 compared to ExoNull. The pronounced cardioprotective effects were abrogated by miR-19a inhibition. Additionally, phosphatase and tensin homolog (PTEN, a predicted miR-19a target) expression was significantly reduced in ExoGATA-4-treated cardiomyocytes, leading to the activation of the Akt and ERK signaling cascades. These findings imply that ExoGATA-4 exosomes are instrumental in conferring cardioprotection through the modulation of specific miRNAs and associated signaling pathways (Zhao et al., 2015; Yu et al., 2015).

#### 3.7.4 Pro-angiogenic

Activation of AKT is known to promote stem cell-mediated cardioprotection; thus, hUCMSC-Exos with AKT overexpression showed enhanced cardioprotective and pro-angiogenic effects. These AKT-enriched exosomes markedly enhance endothelial cell migration, proliferation, and tube formation *in vitro*, as well as neovascularization *in vivo*. Additionally, they exhibit significantly elevated levels of platelet-derived growth factor-D (PDGF-D), which is critical for angiogenesis (Ma et al., 2017a). They also accelerate wound healing and promote angiogenesis by stimulating the Wnt4/β-catenin signaling pathway in endothelial cells (Zhang et al., 2015).

As noted earlier, MSCGATA-4 with elevated GATA4 expression promotes angiogenesis in ischemic myocardium and markedly improves cardiac function (Yu et al., 2013b). Direct intramyocardial injection of ExoGATA-4 at the edge of an ischemic area, following ligation of the left anterior descending coronary artery, substantially improved cardiac contractile function and decreased infarct size (Zhao et al., 2015; Yu et al., 2015). As highlighted in Figure 3, MiR-1246, identified in hUCMSC-Exos, enhances angiogenesis in HUVEC cells by modulating the serine protease 23/snail family transcriptional repressor/alpha-smooth muscle actin (PRSS23/Snail/α-SMA) signaling pathway (Wang et al., 2021d). Similarly, UCMSC-Exos, ADMSC-Exos, and BMMSC-Exos have demonstrated efficacy in a murine MI model, while promoting cardiomyocyte apoptosis and augmenting angiogenesis through elevated levels of hepatocyte growth factor (HGF), angiogenic fibroblast growth factor-β, and vascular endothelial growth factor (VEGF) (Xu et al., 2020).

### 3.8 Other sources of MSCs

MSCs are widely recognized for their therapeutic potential. While umbilical cord, bone marrow, and adipose tissue are commonly cited sources, other notable sources exist that can be utilized for exosome therapy and repair after MI. These alternative sources expand the possibilities for exosome-based therapies across various clinical settings. Studies related to the role of these cells in heart tissue repair are listed in Table 1.

Human amniotic fluid MSC-Exos (hAFMSC-Exos) enhance angiogenesis by increasing the expression levels of hypoxia-inducible factor 1-alpha (HIF-1α) and VEGF in rats with isoproterenol-induced cardiac fibrosis (Hu et al., 2021). HAFMSC-Exos represent a promising therapeutic approach for cardiac fibrosis due to their proangiogenic effects on endothelial cells, which enhance angiogenesis in this condition. For example, studies show that hAFMSC-Exos significantly enhance the motility and migration of HUVECs following oxygen and glucose deprivation (OGD) compared with control group. Moreover, hAFMSC-derived exosomes play a pivotal role in reducing the severity of cardiac fibrosis while simultaneously decreasing collagen I and α-SMA protein levels. They also upregulate CD31 expression in rat models, resulting in increased regenerated microvessels and markedly enhanced angiogenesis following cardiac fibrosis. Importantly, the expression levels of HIF-1α and VEGF are also significantly increased (Hu et al., 2021).

Exosomes derived from human placental mesenchymal stem cells (hPMSCs-Exos) may also be effective in treating cardiac fibrosis after MI. Studies showed that treatment with hPMSCs- Exos considerably improves myocardial fibrosis and left ventricular remodeling compared with the MI cohort. Moreover, administration of hPMSCs-Exos significantly diminishes concentration of molecular biomarkers associated with MI, including AST, BNP, MYO, Tn-I, and TC. It also reduces pro-inflammatory biomarkers such as IL-1β, IL-6, TNF-α, and MCP-1, while elevating high-density lipoprotein (HDL) levels relative to the MI group (Yang et al., 2022b).

Another source of MSCs for cardiac repair after MI is endometrial-derived mesenchymal stem cells (EnMSCs). Endometrium- and menstrual fluid–derived MSC-like populations (EndSCs/MenSCs) represent minimally invasive sources of mesenchymal stem cells. These cells are increasingly recognized as phenotypically heterogeneous. Beyond the International Society for Cell and Gene Therapy (ISCT) minimal MSC markers (CD73, CD90, CD105), endometrial MSCs have been reported to express additional mesenchymal/perivascular and adhesion-associated markers such as CD13, CD29, CD44, CD146, and CD166, while lacking hematopoietic/endothelial markers including CD31, CD34, and CD45 (Vaiciuleviciute et al., 2025; Sepehri et al., 2025; Valatkaitė et al., 2021). In some contexts, pluripotency-associated transcripts (OCT4, SOX2, NANOG, KLF4) have also been detected, further underscoring their heterogeneity. Given the dynamic regenerative nature of the endometrium, source- and subpopulation-dependent differences in secretome and EV cargo may plausibly translate into variable tissue responses, including pro-remodeling or proliferative effects (Skliutė et al., 2021). Research has shown that EnMSCs contribute to myocardial preservation and promote microvascular regeneration. A potential mechanism is the elevated expression and delivery of exosomal miR-21, which appears to be a key factor in the superior paracrine effects of EnMSCs compared with other MSC sources. Exosomal miR-21 secreted from EnMSCs can be transferred to recipient cardiomyocytes and endothelial cells, where it modulates apoptosis and angiogenesis within cardiomyocytes and HUVECs, thereby improving cardiac function post-MI. miR-21 promotes cell survival through the PTEN/Akt signaling pathway, which in turn decreases PTEN expression and increases Akt phosphorylation in recipient cells subjected to exosome treatment, thereby providing a molecular foundation for the amplified paracrine and cardioprotective effects attributed to EnMSCs (Wang et al., 2017a). In another experiment, EnMSC-Exos were shown to prohibit H2O2-induced apoptosis in H9c2 cells and to promote angiogenesis in HUVEC cells, also exerting cytoprotective effects through the epidermal growth factor (EGF)/phosphoinositide 3-kinase (PI3K)/AKT signaling pathway (Yan et al., 2024a). In another study, utilizing EnMSC-Exos in a MI model showed higher histological scores in the EnMSC-Exos group compared with both the untreated MI and sham groups; on day 30, infarct size correlated with the highest histological score in the EnMSC-Exos group relative to the sham and MI groups (Sepehri et al., 2025). As can be seen in Table 1, all these findings suggest that treatment with EnMSC-Exos significantly reduces fibrosis, enhances angiogenesis, and prevents cardiac remodeling, ultimately resulting in improved cardiac function.

### 3.9 Comparative evaluation of MSC sources for exosome-based therapy in MI

Source selection is a determinant of both therapeutic potency and manufacturability in MSC-Exos–based MI repair (Willis et al., 2017). A rigorous comparison should therefore consider: (1) tissue accessibility and donor burden, (2) scalability/exosome yield and expansion capacity, (3) immunogenicity and feasibility of allogeneic use, and (4) MI-relevant functional potency (angiogenesis, anti-apoptosis, anti-fibrosis, and immunomodulation), together with (5) batch variability and senescence-related risks (Elahi et al., 2020; Ma et al., 2024; Han et al., 2022; Moghadasi et al., 2021).

BMMSC-Exos represent the historical “gold standard,” with the most extensive preclinical data supporting their robust immunomodulatory, pro-angiogenic, anti-fibrotic, and anti-apoptotic capabilities (Ahmed et al., 2024). Characteristic miRNAs such as miR-21-5p (for M2 polarization) and miR-25-3p (anti-apoptosis) underpin these therapeutic effects (Palamà et al., 2023; Ryu et al., 2020). However, bone marrow harvest is invasive, and BMMSC expansion and exosome productivity may be constrained by donor-related factors and limited proliferative capacity, which can affect scalability (Lakshman Santra et al., 2017; Dufrane, 2017).

ADSC-Exos offer practical advantages in accessibility, as adipose tissue can be obtained with relatively minimally invasive procedures, and ADSCs typically exhibit robust *in vitro* growth and exosome production (Rau et al., 2025). Their therapeutic profile is potent and often comparable to BMMSCs, facilitated by mechanisms including miR-31 and miR-125a for angiogenesis and miR-146a for anti-apoptosis (Pan et al., 2019; Xie et al., 2017a; Gwiggner et al., 2014). Consequently, ADSCs often represent a strong “balance point” between feasibility and potency for scalable exosome manufacturing (Chen et al., 2022a). However, their limitations include: Donor metabolic status (e.g., obesity, diabetes) can alter exosome cargo and reduce therapeutic potency (Chen et al., 2025). Standardization of donor selection remains a challenge.

HUCMSCs emerge as a particularly promising source. Sourced from medical waste tissue, their harvesting entails no invasiveness or ethical concerns (Li et al., 2022). HUCMSCs are often reported to exhibit higher proliferative capacity and scalable manufacturing potential, likely due to their neonatal origin (Wang et al., 2018). Their potent effects are driven by miRNAs like miR-19a (anti-apoptosis) and miR-125b-5p (anti-fibrosis *via* the Smad7 axis) (Huang et al., 2020; Wang et al., 2018). Low immunogenicity further supports HUCMSCs as a leading candidate for allogeneic, scalable MSC-Exos production for MI applications (Ding et al., 2025).

As summarized in Table 2, BMMSC-Exos remain the most extensively studied benchmark source, while ADSC-Exos provide a practical balance between accessibility and therapeutic potency, and hUCMSC-Exos stand out for their non-invasive sourcing, neonatal proliferative advantage, and low immunogenicity. Each source offers distinct strengths and limitations for MI repair, underscoring the need for harmonized reporting of expansion kinetics, exosome yield, standardized characterization, and MI-relevant potency assays. Future studies should also implement stringent release criteria to minimize variability and enable rational source selection.

**TABLE 2:** Comparative analysis of primary MSC sources for exosome production in MI therapy.

Feature	BMMSCs	ADSCs	HUCMSCs
Invasiveness of harvesting	High (painful bone marrow aspiration)	Low (minimally invasive liposuction)	None (medical waste tissue)
Cell and exosome yield	Moderate	High	Very High
Proliferation and expansion capacity	Moderate	High	Very High
Immunomodulatory potential	Well-established, strong evidence	Strong, comparable to BMMSCs	Potent, often superior due to neonatal source
Pro-angiogenic potential	Strong, well-documented (e.g., *via* miR-210, miR-132)	Strong, well-documented (e.g., *via* miR-31, miR-125a)	Very strong, enhanced by factors like AKT-overexpression
Anti-fibrotic evidence	Strong (e.g., *via* miR-212-5p, modulation of TGF-β/Smad)	Strong (e.g., *via* miR-671 targeting TGFβR2/Smad2)	Strong (e.g., *via* miR-125b-5p/Smad7 axis)
Anti-apoptotic Evidence	Extensive (e.g., *via* miR-21a-5p, miR-144, PTEN/PI3K/Akt)	Strong (e.g., *via* miR-146a, miR-214)	Extensive and potent (e.g., *via* miR-19a, GATA-4 engineered exosomes)
Key advantages	• “Gold standard,” most extensively studied • Robust, well-characterized preclinical data	• High accessibility and yield • Excellent proliferation • Strong therapeutic profile	• Highest proliferative capacity • Low immunogenicity • Potent paracrine activity • No ethical concerns
Characteristic miRNAs/mechanisms	MiR-21-5p (M2 polarization), miR-125b (anti-apoptosis/autophagy), miR-182 (anti-inflammation), miR-25-3p (anti-apoptosis)	MiR-31 (angiogenesis *via* FIH1/HIF-1α), miR-125a (angiogenesis *via* DLL4 inhibition), miR-146a (anti-apoptosis)	MiR-19a (anti-apoptosis *via* SOX6), miR-24 (anti-apoptosis/anti-inflammation), miR-181a (immunomodulation), miR-125b-5p (anti-fibrosis *via* Smad7)

Abbreviations: AKT, protein kinase B; TGF-β, Transforming growth factor β; PI3K, phosphoinositide 3-kinase; PTEN, phosphatase and tensin homolog; FIH1, factor inhibiting; HIF-1; alpha, hypoxia-inducible factor 1-alpha.

### 3.10 Comparison of mesenchymal stem cells with other cellular sources for cardiac repair

Research findings strongly indicate that MSCs, a specific type of adult stem cells, confer substantial benefits in the context of cardiac repair and regeneration. Their remarkable accessibility, favorable safety profile, and significant paracrine effects facilitate tissue healing and functional recovery. Nevertheless, outcomes associated with the utilization of MSCs can differ considerably across various studies, reflecting the complexity of biological responses and experimental conditions (Yan et al., 2024b).

ESCs and induced iPSCs represent highly promising cell sources for cardiac regenerative therapy, particularly in the context of MI (Lee et al., 2017; Sugiura et al., 2024). Both human ESC-derived cardiomyocytes (ESC-CMs) and iPSC-derived cardiomyocytes (iPSC-CMs) have demonstrated the ability to improve cardiac function in models of myocardial infarction (Tachibana et al., 2017; Chen et al., 2014). Studies have shown that ESC-CMs and iPSC-CMs can facilitate comparable cardiac repair (Lee et al., 2017). For instance, sub-acute transplantation of both ESC-CMs and iPSC-CMs into rats post-MI improved cardiac performance, accompanied by increased angiogenic gene expression under hypoxic conditions (Lee et al., 2017). At a single-cell level, both cell types exhibited comparable calcium handling and electrophysiological properties, with gene expression patterns resembling those of the human fetal heart (Chen et al., 2014).

Differentiation protocols have been established to generate cardiomyocytes from human pluripotent stem cells (Lian et al., 2012). Moreover, human cardiac extracellular matrix supports ESC and iPSC proliferation and promotes cardiac lineage commitment, suggesting potential for guiding differentiation toward cardiomyocyte phenotypes for myocardial repair (Oberwallner et al., 2015). iPSCs offer a distinct advantage over ESCs due to their potential for autologous transplantation, thereby reducing the risk of immune rejection and circumventing ethical concerns associated with embryonic cells (Ali et al., 2025). Their unlimited proliferative capacity and ability to differentiate into cardiomyocytes further underscore their therapeutic promise (Sugiura et al., 2024).

Despite encouraging preclinical and early clinical results, the full therapeutic potential of stem cell therapy for ischemic heart disease remains unrealized. Challenges include poor cell survival, limited differentiation efficiency, and risks such as tumorigenicity. Genetic engineering approaches are being explored to enhance reparative capacity, while heterogeneity of clinical trials, small sample sizes, and short follow-up durations limit the generalizability of current findings (Ali et al., 2025; Lee et al., 2017). Future advancements may involve the integration of regenerative treatments like extracellular vesicle therapy and the use of biomaterials to engineer stem cells for reduced immunogenicity and improved survival and integration within the heart (Sugiura et al., 2024).

Endothelial progenitor cells (EPCs) also play a crucial role in promoting vascular repair and neovascularization. However, their limited ability to regenerate cardiac muscle tissue restricts their utility as a primary therapeutic option, positioning them instead as complementary to other stem cell types with direct cardiomyogenic potential (Esquiva et al., 2018).

In conclusion, MSCs currently represent a more feasible and pragmatic option for cardiac repair initiatives, given their accessibility, safety, and paracrine-mediated benefits. Nevertheless, pluripotent stem cells (ESCs and iPSCs) provide superior regenerative potential, albeit with ethical and safety challenges, while EPCs contribute primarily to vascular repair. Synergistic approaches that combine different stem cell types may ultimately help overcome current limitations and controversies surrounding safety and efficacy in cardiac regenerative therapies.

## 4 Biomaterial-based MSC-derived exosomes for MI regeneration

**FIGURE 4:**
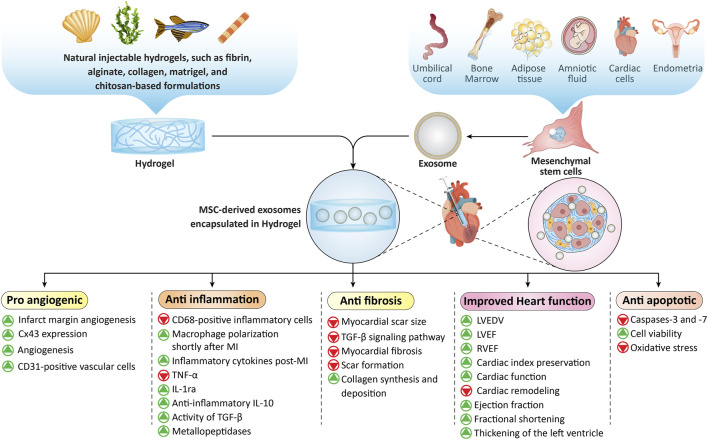
Schematic representation of the molecular processes by which HUCMSC-Exos mod-ulate cardiac injury and repair after MI. HUCMSC-Exos trigger anti-inflammatory effects by promoting M2 macrophage polarization and suppression of pro-inflammatory cytokines by miRNAs such as miR-181a, miR-19a, miR-100-5p, and miR-24, thereby influencing fibroblast dif-ferentiation and suppressing inflammasome activation (NLRP3). They also promote cell survival by regulating apoptosis-related signaling pathways including PTEN/AKT/ERK, JNK3/caspase-3, Bax, TIMP2, and AMPK/mTOR through miR-19a and miR-24 activities. Oxidative stress is attenuated by reducing ROS and inhibiting iron deposition and lipid peroxidation markers such as malondial-dehyde. Furthermore, HUCMSC-Exos stimulate angiogenesis by delivering pro-angiogenic factors and miRNAs including VEGF, FGF-β, HGF, PDGF-D, Wnt4/β-catenin, and miR-1246, regulating targets such as PRSS23, Snail, and α-SMA to promote vascular regeneration, collectively supporting inflammation resolution, apoptosis inhibition, oxidative-stress reduction, and angiogenesis en-hancement for tissue repair. IL-10, Interleukin-10; TNF-α, Tumor Necrosis Factor-alpha; IL-4, Interleukin-4; IL-1β, Interleukin-1 beta; CXCL9, C-X-C motif chemokine ligand 9; VEGF, Vascular Endothelial Growth Factor; LVEDV, Left Ventricular End-Diastolic Volume; LVEF, Left Ventricular Ejection Fraction; RVEF, Right Ventricular Ejection Fraction. “Created with Adobe Illustrator 27.6.1”.

Since exosomes are rapidly cleared from the bloodstream after entering circulation, leading to their swift elimination from blood vessels and subsequent uptake by parenchymal organs (Schiffelers et al., 2000; Imai et al., 2015). Research indicates that exosomes have a remarkably short plasma half-life, ranging from approximately 2–4 min (Saunderson et al., 2014). Moreover, studies have shown that exosomes can accumulate in the liver, spleen, lungs, and gastrointestinal tract within as little as 2 h after systemic administration (Takahashi et al., 2013; György et al., 2015). This process largely results from the phagocytic function of macrophages within the liver and spleen (Imai et al., 2015). This effect is thought to be associated with the exposure of phosphatidylserine on exosomes, facilitating their recognition and subsequent phagocytosis by macrophages (Cocco and Ucker, 2001). In the heart, rapid blood flow may hinder EVs accumulation within the organ. Intravenously injected exosomes have a blood circulation half-life of approximately 2 min, with minimal detection observed 4 h post-injection (Takahashi et al., 2013). Consequently, due to their inherent instability, variable delivery rates, and the presence of reactive phagocytic cells during cardiac tissue healing, exosomes are often cleared from target sites (Zou et al., 2021; Zhang et al., 2018; Ma et al., 2016; Kim and Mok, 2019). Therefore, establishing approaches that ensure the sustained presence of EVs at the intracardiac injection site is a prerequisite for their clinical application. Another consideration is the uptake capacity of target cells (Tian et al., 2010). Thus, achieving both stability and retention is essential for effective *in vivo* exosome application to obtain enhanced regenerative outcomes. The approach involves designing and utilizing exosome carriers with structural and biological characteristics tailored to the specific cell type and intended application. As is apparent in Figure 4, this can be accomplished by integrating exosomes with scaffolds that act as carriers, aiming to enhance their functionality and efficacy (Riaud et al., 2020). A wide variety of scaffolds has been developed from both natural and synthetic biomaterials. Natural scaffolds provide intrinsic biological cues but often require complex procedures to ensure reproducibility and purification. Conversely, synthetic scaffolds benefit from standardized manufacturing protocols but typically require functionalization to deliver the essential biological signals to endogenous or transplanted cells (Patel and Patel, 2024). When selecting a scaffold for tissue regeneration, several critical parameters must be considered, including biodegradability and biocompatibility. Furthermore, a localized delivery approach for these biodegradable scaffolds is essential to minimize systemic side effects. The delivery method should also be simple and rapid, considering the heart’s continuous contractile activity. It is also crucial that these scaffolds are non-cytotoxic and biocompatible to ensure a beneficial effect with minimal or no immunological response (Karam et al., 2012; Christman and Lee, 2006; Rane and Christman, 2011). Additionally, a localized delivery is vital to mitigate systemic side effects, and the administration method should be straightforward and rapid given the myocardium’s continuous contraction (Roacho-Pérez et al., 2022).

In the domain of cardiac regeneration, scaffold selection is critically governed by the heart’s distinctive contractile properties. To address these requirements, hydrogels with favorable biomechanical and elastic characteristics are commonly employed. These materials not only prepare structural support but also contribute significantly to the partial restoration of impaired cardiac function (Mantha et al., 2019; Mathieu et al., 2012). A variety of biomaterials have been investigated for hydrogel fabrication in tissue engineering, including ceramics as well as natural and synthetic polymers (O’brien, 2011). Ceramics, characterized by relatively low elasticity and high mechanical strength, are primarily used in the repair and regeneration of bone and dental tissues. In contrast, natural and synthetic biomaterials are preferred for regeneration of soft tissues, including cardiac and neural tissues (Mazzoni et al., 2021). Natural biomaterials closely mimic the characteristics of native tissues; however, they often exhibit batch-to-batch variability owing to the heterogeneity of their natural sources. Synthetic biomaterials overcome this limitation through controlled and customizable artificial synthesis processes (Hernandez and Christman, 2017). In preparing targeted exosome-loaded hydrogels, careful evaluation of parameters such as swelling rate, surface charge, porosity, and degradation kinetics is essential, as these factors directly affect exosome encapsulation and release. The porous and loosely organized network of hydrogels is crucial for ensuring efficient loading and sustained exosome delivery (Raina et al., 2022). Recently, hydrogel-based systems designed for exosome delivery have become a major focus of strategies aimed at repairing and improving cardiac tissue function after MI. (Wang et al., 2023b). This innovative delivery approach involves encapsulating EVs within hydrogels, which effectively prolongs their retention and uptake in cardiac tissue. Additionally, hydrogels possess the unique ability to continuously release EVs, further supporting cardiac repair (Zhang et al., 2021b; Keith et al., 2014; Xie et al., 2022). The comparative applications of biomaterial scaffolds combined with exosomes—including scaffold type, source, model and time, dose and route, and therapeutic outcomes—are summarized in Supplementary Table S1, underscoring their role in improving exosome stability, retention, and regenerative efficacy. The process of encapsulating exosomes within bulk biomaterials or within a porous network during fabrication provides an effective alternative to surface modification, as it helps preserve the bioactivity of the exosomes (Rahmati et al., 2023; Czosseck et al., 2022). By employing this technique, exosome release can be both spatially controlled and temporally sustained. Crosslinking of hydrogel precursors loaded with exosomes leads to uniform matrix integration, followed by gradual liberation of exosomes through diffusion and enzymatically- or hydrolytically-mediated degradation pathways. Moreover, exosome therapeutics can be encapsulated within microscale or nanoscale carriers composed of lipids, polymers, or organic matrices, permitting environmentally triggered and temporally regulated release of their cargo. Porous three-dimensional scaffolds not only permit efficient loading of exosomes but also ensure their sustained elution upon integration within tissue defect sites, thereby enhancing regenerative outcomes. This encapsulation not only provides protection but also prolongs exosome stability and release, overcoming limitations related to their short half-life and instability (Rahmati et al., 2023). Moreover, scaffolds serve as structural templates, supporting tissue regeneration precisely at damaged sites. Despite progress to date, achieving precise release kinetics, efficient cargo loading, and robust storage stability still requires ongoing refinement in biomaterial engineering approaches. Additionally, rigorous *in vivo* functional studies in clinically relevant models are needed to fully characterize exosome bioactivity (Hazrati et al., 2023; Murali and Holmes, 2021; Zheng et al., 2023). Cutting-edge biomaterial strategies and cell-free therapeutic modalities collectively demonstrate the considerable promise of exosomes in promoting tissue regeneration. Hydrogels with controllable degradation properties are particularly advantageous for safeguarding exosomes during the delivery process (Cao et al., 2021). These materials exhibit biocompatibility, hydrophilicity, compositional adaptability, injectability, electrical conductivity, and tunability, making them well-suited for replicating the structural and functional characteristics of the cardiac ECM. Such properties create an extracellular environment conducive to cell adhesion, proliferation, and migration both *in vitro* and *in vivo* (Foster et al., 2017; Bose et al., 2018). Moreover, hydrogels offer an elegant delivery system that can be adjusted according to the substrates used in their design. They have been extensively applied in a range of therapeutic strategies for cardiac tissue regeneration (Li et al., 2023c). These hydrogels can be delivered either as injectable formulations directly into the injured myocardium or *via* coronary artery perfusion (Yu et al., 2022). Natural injectable hydrogels, including fibrin, alginate, collagen, Matrigel, and chitosan-based formulations, have been widely studied for applications in cardiac tissue engineering (Segers and Lee, 2011; Wang and Guan, 2010). An oxygen-generating, antioxidant, nanofibrous bi-layered cardiac patch (PUAO-CPO-Collagen), electrospun over a porous collagen scaffold and supplemented with ADSC-Exos, was developed to optimize cardiac regeneration. *In vitro* assays demonstrated that the PUAO-CPO-Collagen patch, in conjunction with ADSC-Exos, exerted pro-survival, proliferative and, pro-angiogenic effects. In a rat MI model, implantation of the bi-layered patch substantially improved cardiac function, diminished fibrotic scar deposition, and attenuated adverse ventricular remodeling through augmented angiogenesis and reduction of oxidative stress (Shiekh et al., 2022). The delivery of MSC-Exos through an alginate hydrogel mitigated cardiomyocyte apoptosis and induced macrophage polarization in the early post-MI period, while simultaneously enhancing long-term cardiac outcomes. Importantly, EVs incorporated within the hydrogel demonstrated prolonged cardiac residency, contrasting sharply with the rapid clearance observed when EVs were administered without a hydrogel carrier (Lv et al., 2019). An injectable, oxygen-releasing, bio-macromolecular hydrogel system was developed by integrating catalase (CAT)-loaded alginate and fibrin with MSC-derived exosomes. *In vitro* studies demonstrated that the gold nanoparticle-enhanced composite hydrogel (Exo/Hydro/AuNPs/CAT) possessed electrical conductivity comparable to that of native cardiac tissue and facilitated efficient CAT release. The hydrogel sustained oxygen delivery for nearly 5 days under hypoxic conditions and, after 7 days of culture, secreted paracrine factors comparable to those of rat neonatal cardiomyocytes, cardiac fibroblasts, and HUVECs, thereby mimicking capillary structure and function. In a rat MI model, *In vivo* application of this injectable conductive hydrogel markedly attenuated left ventricular remodeling and myocardial dysfunction, enhanced angiogenesis at the infarct margin, reduced cardiomyocyte apoptosis and necrosis, and upregulated Connexin43 (Cx43) expression (Xu et al., 2025). Recent investigations have demonstrated that the co-delivery of cardiac cell-laden calcium alginate microgels and MSC-Exos represents a promising strategy for improving myocardial regeneration following infarction. The co-delivery approach significantly improved echocardiographic outcomes, cardiac-specific gene expression, and biomarker levels relative to groups receiving only exosomes or cardiac cells, both of which showed minimal myocardial regeneration (Banikarimi et al., 2025). ADSC-Exos were encapsulated within a bioengineered injectable hydrogel composed of alginate, collagen, and calcium gluconate. This hydrogel system facilitated prolonged myocardial retention and spatially uniform release of exosomes following intramyocardial injection in female rats. The exosome-hydrogel group achieved maximal cardiac retention, whereas direct administration of unencapsulated exosomes led to their systemic distribution and accumulation in non-target organs, including the spleen and liver (Gil-Cabrerizo et al., 2022).

A study investigated the use of self-assembling peptide amphiphiles (PA) containing cardiac protective sequences (GHRPS) and a degradable motif (GTAGLIGQ) for the delivery of xenogeneic hUCMSC-Exos in a rat MI model. Administration into the peri-infarct area significantly attenuated fibrotic remodeling, decreased TGF-β1 expression, reduced cardiomyocyte apoptosis, enhanced CD31-positive vascular cell density, and lowered CD68-positive inflammatory cell presence (Han et al., 2019).

A pivotal porcine MI study delivered cardiac ADMSC-Exos using a decellularized pericardial scaffold embedded with peptide hydrogel. This approach improved cardiac function, evidenced by increased in left ventricular end-diastolic volume (LVEDV), left ventricular ejection fraction (LVEF), maintenance of cardiac index, improvements in right ventricular ejection fraction (RVEF), reductions in myocardial scar size and adverse remodeling and, no induction of left ventricular hypertrophy. Notably, administration of cardiac ADMSC-Exos within a decellularized pericardial scaffold modulated both systemic and local inflammatory and fibrotic mediators. At the molecular level as can be seen in Figure 4, cardiac ADMSC-Exos reduced TNF-α, increased IL-1ra (the natural IL-1 receptor antagonist), and enhanced local IL-10 expression. These changes regulated downstream profibrotic pathways, including TGF-β, metallopeptidases, and related mediators, culminating in attenuated collagen synthesis and deposition (Monguió-Tortajada et al., 2022).

Researchers engineered elastic cryogels by modifying polyurethane with the antioxidant gallic acid (PUGA) and subsequently coating them with decellularized extracellular matrix (dECM) to enhance adhesiveness, biocompatibility, and hemocompatibility. The PUGA-dECM + EXO composite, incorporating ADMSC-Exos, was tested in a rat MI model. Echocardiographic analysis 8 weeks post-implantation revealed significant functional recovery in the treatment group. Histological examination demonstrated reduced fibrosis and enhanced angiogenesis, while immunostaining indicated decreased oxidative stress (Das et al., 2024). A novel hydrogel incorporating MSC-Exos and selenium nanoparticles (Se NPs) within a collagen type I (COL-I)/tannic acid (TA) matrix was developed for AMI treatment. *In vitro* analyses revealed enhanced cell viability, as confirmed by MTT and LDH assays, indicating the hydrogel’s biocompatibility for AMI therapy. Migration assay demonstrated approximately 95% migration of cardiac endothelial cells (cECs) in the Se-Exos/hydrogel group compared with controls. Additionally, apoptosis assay showed that the Se-Exos/hydrogel significantly mitigated LPS-induced cell apoptosis relative to other groups. *In vivo*, injection of the Se-Exos/hydrogel into AMI rat models improved cardiac function, increased left ventricular (LV) wall thickness and and markedly reduced infarct size *versus* untreated controls (Lin et al., 2023). Islet-1 (ISL1) overexpression has been shown to enhance the paracrine functions of MSCs and promote angiogenesis in MI models. To leverage these effects, genetically engineered ISL1-MSC-Exos were administered using an angiogenin-1 hydrogel (Ang-1 gel). This strategy not only increased the retention of ISL1-MSC-Exos but also amplified their anti-apoptotic, proliferative, and angiogenic activities in endothelial cells. Echocardiographic evaluations demonstrated that the Ang-1 gel significantly enhanced the therapeutic effects of ISL1-MSC-Exos in MI. Collectively, these findings suggest that ISL1-MSC-Exos possess endothelial-protective and pro-angiogenic properties; Ang-1 gel improves retention at ischemic sites, promoting endothelial survival, angiogenesis, and myocardial repair post-MI (Hu et al., 2022). HIF-1α–expressing MSC-Exos encapsulated in an arginine-glycine-aspartate (RGD)-biotin hydrogel facilitated uptake by human endothelial cells and rat cardiomyocytes. In rat cardiomyocytes, RGD-biotin hydrogel–HIF-1α-Exos reduced caspase-3/7 activity under normoxic and hypoxic conditions. Following this treatment human ECs formed more lumens *In vitro*. *In vivo*, RGD-biotin hydrogel–HIF-1α-Exos increased ejection fraction and fractional shortening, and reduced type I collagen deposition, with greater left ventricular wall thickening (Wang et al., 2021a) (Figure 4).

A gelatin-based biocompatible microneedle (MN) patch was engineered to deliver HUCMSCs-derived exosomes containing the Exo/miR-29b mimic for preventing excessive cardiac fibrosis in MI therapy. This innovative platform combines the benefits of microneedle patch, exosomes, and microRNAs. After implantation into the infarcted myocardium, the MN patch maintained a higher local concentration of Exo/miR-29b mimic and facilitated prolonged exosome release as the microneedles gradually dissolved. The released exosomes were taken up by cardiac fibroblasts, leading to upregulation of miR-29b, inhibition of the TGF-β signaling pathway, and prevention of myocardial fibrosis through modulation of ECM remodeling. In mouse models, application of this composite patch markedly attenuated inflammation, left ventricular wall thinning, infarct size, and fibrosis in the affected region, thereby promoting cardiac repair after MI. Collectively, this delivery system shows substantial potential as a therapeutic strategy for MI (Yuan et al., 2023).

A composite injectable hydrogel was developed by integrating curcumin (Cur), exosomes derived from BMMSCs, and decellularized porcine cardiac extracellular matrix (dECM) hydrogels, to confer antifibrotic properties for the treatment of MI. Curcumin was preloaded into exosomes *via* electroporation, enabling the hydrogel system to deliver curcumin-encapsulated exosomes to attenuate cardiac fibrosis following MI. Both exosomes and dECM hydrogels exhibit intrinsic bioactive properties that support cardiac repair while enhancing the solubility and sustained release of curcumin within the infarcted myocardium. *In vitro* studies confirmed that Exos/Cur were taken up by fibroblasts, thereby preventing their transformation into myofibroblasts and suppressing fibrotic progression. *In vivo* administration of the composite hydrogel in mice with MI promoted angiogenesis, increased left ventricular wall thickness, reduced infarct size, and inhibited fibrosis, collectively facilitating myocardial repair. This approach effectively combines the therapeutic advantages of natural biomaterials (dECM), EVs (exosomes), and an antifibrotic agent (Cur), offering a promising strategy for the clinical management of myocardial fibrosis after MI (Wang et al., 2023c). A novel hydrogel system, designated HAD + Exos, was engineered by encapsulating cardiomyocyte exosomes derived from induced pluripotent stem cells (iCM-Exos) within asymmetric hyaluronic acid-g-(2-aminoethyl methacrylate hydrochloride–dopamine) (HAD) hydrogels. This platform was specifically designed to alleviate oxidative stress and prevent pericardial adhesion following cardiac surgery. The asymmetric configuration of the hydrogel ensures firm adhesion to the myocardial surface while simultaneously resisting attachment to the thoracic cavity, thereby limiting macrophage recruitment and preserving the therapeutic activity of iCM-Exos (Wang et al., 2023a).

Therefore, a broad study into the synergistic effects of diverse hydrogels—including stem cell-based, peptide-based, and smart hydrogels—in combination with EVs may provide new insights into how these materials interact during cardiac regeneration (Hashemi et al., 2024). It is essential to tailor the chemical and physical characteristics of these hydrogels and to optimize *in situ* crosslinking techniques to ensure stability and controlled release during application. Such advancements are fundamental for creating more effective strategies for myocardial regeneration, ultimately improving outcomes and quality of life for patients with heart disease (Holme et al., 2025).

Findings further suggest that, beyond the intrinsic therapeutic effects of exosomes, the route of administration critically influences their biodistribution, thereby enhancing therapeutic efficacy against MI-induced cardiac dysfunction. However, as with any therapeutic modality considered for clinical application, certain aspects still require improvement to facilitate the rapid translation of EVs into clinical settings.

## 5 Challenges and future direction

Current treatments for cardiac conditions, while effective at reducing patient mortality, do not significantly regenerate heart tissue. Therefore, it is crucial to continue preclinical studies to enable potential clinical translation (Kou et al., 2022). Exosomes have therapeutic effects that are comparable to, and in some cases greater than, those of their parent cells, which consistently show strong immunomodulatory and cardiac regenerative potential in both preclinical models and clinical studies (Qin et al., 2024). Although adult stem cells can be easily isolated, their limited expansion potential and restricted plasticity hinder their viability as candidates for clinical application. By contrast, exosomes offer an acellular therapy with multiple mechanisms of action and low immunogenicity, making them as a promising alternative to both cellular therapies and pharmacological treatments (Lima Correa et al., 2021).

A major factor underlying the failure of clinical trials is the absence of standardized and rigorous cell recovery protocols, a problem that also affects EVs. Despite the establishment of standardized isolation protocols, considerable variations persist, hindering the clinical application of exosomes (Yadav et al., 2024a). These variations often come from differences in purification methods, which can lead to heterogeneity within exosome populations (Doyle and Wang, 2019).

Several techniques are available for exosome isolation, including ultracentrifugation, size exclusion chromatography, ultrafiltration, and precipitation methods; however, a universally accepted standard protocol has yet to be established. Among these approaches, ultracentrifugation remains the most commonly employed and cost-effective method, yet it has notable limitations. Specifically, it produces a relatively lower yield of exosomes compared to alternative techniques such as size exclusion chromatography and ultrafiltration. Moreover, this method involves multiple procedural steps, making it labor-intensive and time-consuming (Gao et al., 2023). Each method has its own pros and cons. A key challenge in exosome isolation is their heterogeneity and purity, given that current methodologies predominantly rely on size- and density-based separation. Consequently, the isolated fractions may be contaminated with particles of similar size and density, such as microvesicles, small apoptotic bodies, or non-exosomal proteins. Furthermore, variables such as cell culture conditions, including passage number and media composition, the specific isolation protocol employed, and the equipment used, can introduce variability in both the quantity and quality of the obtained exosomes. In summary, issues related to heterogeneity, contamination, low yield, and a lack of standardized protocols significantly hinder the clinical application of MSC-Exo (Patel et al., 2019; Chen et al., 2022b).

Another critical challenge relates to cellular senescence and its impact on the MSC secretome. MSC-Exos are frequently regarded as safer cell-free alternatives; however, their bioactivity is significantly influenced by contextual factors (Yan et al., 2025). Senescence-related regulatory changes can alter EV size and cargo, and senescent EVs may contain unpredictable mixtures of growth factors and cytokines that can promote senescence induction and an inflammation-related “domino effect,” ultimately contributing to tissue inflammation (Smirnova et al., 2023). Moreover, MSCs may develop senescent characteristics during *in vitro* expansion or cryopreservation, with their secretome creating a pro-inflammatory microenvironment affecting surrounding cells (Smirnova et al., 2023). Senescence-associated secretory phenotype (SASP) factors are particularly concerning, as they can induce dysfunction in neighboring cells and trigger their entry into senescence—a “contagious-like” effect. Ageing further exacerbates this risk by increasing stressors and reducing immune clearance, leading to the accumulation of senescent cells within tissues (Aguayo-Mazzucato, 2020). To mitigate these risks, future translational studies should incorporate senescence screening of MSC sources and manufacturing conditions, alongside rigorous EV cargo characterization. Functional assays are also needed to evaluate pro-senescent and pro-inflammatory effects in recipient cells prior to clinical application (Aguayo-Mazzucato, 2020). Nevertheless, MSCs—and especially MSC-Exos—remain promising cardioreparative therapeutic options due to the extreme standardization of EV manufacture and stringent screening of MSC senescence. According to preclinical research, MSC-EVs can reduce infarct size, improve left ventricular function after myocardial infarction, and increase angiogenesis and cardiomyocyte survival (Hassanzadeh et al., 2024).

Another important challenge relates to the heterogeneity of MSCs derived from unconventional sources such as endometrium and menstrual fluid. EndSCs/MenSCs, while minimally invasive, exhibit variable marker expression and secretome profiles that may influence tissue responses. In particular, subpopulation-dependent differences in EV cargo could theoretically amplify proliferative or remodeling signals, raising concerns about endometrial thickening under susceptible conditions (Valatkaitė et al., 2021; Vaiciuleviciute et al., 2025). Accordingly, extended immunophenotyping beyond CD73/CD90/CD105, standardized manufacturing, and functional potency/safety assays are recommended prior to clinical translation, alongside explicit monitoring of endometrial endpoints in preclinical and clinical studies.

Efficient delivery of MSC-Exos to targeted tissue sites, alongside the maintenance of their biological activity, remains a major challenge in their therapeutic application (Zhao et al., 2024). Delivering MSC-Exos to sites of injury while maintaining their therapeutic concentration remains challenging (Gallet et al., 2017). Therefore, the establishment of standardized dosing regimens to sustain therapeutic levels of MSC-Exos in the body is essential. To overcome this challenge, various delivery strategies have been explored with promising results in preclinical models. One potential strategy to mitigate the risks associated with biodistribution is to associate exosomes with scaffolds, which are already established as supportive structures in cell therapy. These strategies encompass the incorporation of MSC-Exos into biocompatible hydrogels, their loading onto scaffolds or microneedle patches, as well as engineering or designing them to enable targeted delivery to specific tissue sites (Chen et al., 2022c). Despite their inherent regenerative properties, the application of exosome-bearing hydrogels is limited by several factors that hinder their effectiveness in ischemic cardiac tissue. The delivery of these hydrogels often necessitates invasive techniques, including complex surgical procedures and direct manipulation of the heart (Radhakrishnan et al., 2014). Consequently, the development of minimally invasive delivery methods is of paramount importance. Cardiac patches composed of natural or synthetic materials can serve as reliable platforms for the efficient administration of exosomes into the infarcted myocardium (Casado Mayo et al., 2023). However, while cardiac patches have traditionally been employed for the delivery of small molecules to targeted cardiac tissue, their clinical translational potential is constrained by the invasive nature of implantation, typically necessitating thoracotomy and suturing to the myocardium (Radhakrishnan et al., 2014). To overcome this limitation, researchers have developed a minimally invasive, sprayable cardiac patch seeded with MSC-Exos. To ensure optimal gelation, a commercially available fibrin sealant was used as the exosome loading vehicle. The exosomes not only enhanced the retention of MSC-Exos on the heart but also mitigated the surgical stress associated with open-chest procedures (Yao et al., 2021). To avoid secondary tissue damage associated with intramyocardial injections, a minimally invasive, injection-free delivery method has been developed. This involves spraying a mixture of exosomes, gelatin methacryloyl precursors, and photoinitiators onto the heart surface, followed by visible light irradiation to form an *in situ* cardiac patch. This technique improves exosome retention and enhances therapeutic efficacy; however, it remains invasive due to the necessity of thoracotomy (Tang et al., 2022).

Furthermore, MSC-Exos, whether delivered in combination with scaffolds or independently, have been extensively studied in both *in vitro* and *in vivo* models, significantly contributing to advancements in myocardial regeneration research. Multiple signaling pathways have been identified, providing insight into the differentiation processes and regulatory mechanisms underlying the downstream effects of these targeted exosomes. Irrespective of the cell type, exosome subtype, target molecules, or delivery method, thorough characterization and rigorous experimental evaluation are crucial for improving our understanding of the successful clinical translation of regenerative strategies aimed at preserving myocardial tissue (Wu et al., 2018). The majority of current research has concentrated on elucidating how exosomal contents influence signaling pathways involved in processes such as cell death, angiogenesis, and inflammation. However, the detailed molecular mechanisms, particularly those acting upstream of these cascades, are still insufficiently understood. Presently, unlike single-cell analytical techniques, there is a lack of suitable methodologies for the analysis of individual exosomes. Investigating the cargo of single exosomes could offer deeper insights into the heterogeneity within the exosome population and potentially reveal critical aspects of their molecular mechanisms (Boriachek et al., 2018).

Taken together, these studies indicate that the controlled, gradual release of exosomes can confer therapeutic benefits while limiting their undesired biodistribution throughout the body. The evidence further suggests that exploring a range of scaffold materials may allow the therapeutic potential of exosomes to be harnessed more effectively than traditional cell-based approaches. Investigating the synergistic interactions between exosomes and scaffolds as a strategy for cardiac repair represents a promising avenue for future research.

Nevertheless, additional investigations, particularly preclinical and clinical studies, are essential to establish the safety and efficacy of this approach.

## 6 Conclusion

MSC-Exos have emerged as a novel and promising therapeutic strategy for myocardial infarction, providing advantages over conventional stem cell transplantation, including enhanced safety and targeted paracrine effects. Their capacity to carry and deliver a diverse range of bioactive molecules, including proteins, lipids, and miRNAs, enables MSC-Exos to modulate critical processes involved in cardiac repair, such as angiogenesis, anti-apoptotic signaling, and anti-inflammatory responses. Given their potential significance in injury response, tissue repair, and remodeling, numerous studies, as well as preclinical and clinical trials, are currently evaluating MSC-Exos for tissue regeneration. In particular, the roles of MSC-Exos derived from sources such as BMMSCs, ADSCs, and HUCMSCs are being elucidated from multiple perspectives to optimize their therapeutic utility in MI. Despite substantial progress in understanding their mechanisms of action, clinical translation faces challenges related to delivery, stability, and scalability. Advances in biomaterials, especially hydrogel systems, hold great promise to enhance exosome retention and efficacy at injured sites. Moving forward, further research into exosome potency, delivery strategies, and long-term safety will be vital to fully harness their regenerative potential. The integration of exosome biology with advanced materials science paves the way for next-generation regenerative therapies, which have the potential to substantially improve clinical outcomes for patients with myocardial infarction.
