# Supplementary material for: Mesenchymal stem cell-derived exosomes in myocardial infarction repair: therapeutic potential and scaffold-based delivery strategies
Source: Front Pharmacol. 2026 Feb 5;17:1762630. doi: 10.3389/fphar.2026.1762630 (PMC12916389; doi:10.3389/fphar.2026.1762630)
Supplement: Supplementary file 1 [file Table1.docx]

**Supplementary Table 1.** Biomaterial Scaffolds Combined with Exosomes for Cardiac Repair.

| Biomaterials scaffold | Source | Model and time | Dose and route |  | Results | Ref. |
| --- | --- | --- | --- | --- | --- | --- |
| Gelatin microneedle (MN) patch | HUCMSC-Exos (miR-29b mimic) | Mouse MI model | Implanted in infarcted area |  | Reduced inflammation, infarct size, LV wall thickness, fibrosis; inhibited TGF-β signaling via miR-29b | (Yuan et al., 2023) |
| Injectable hydrogel (dECM + alginate) | BMSC-Exos + Curcumin | Mouse MI model | Injected into myocardium |  | Prevented fibroblast transformation; promoted angiogenesis; reduced infarct size; inhibited fibrosis | (Wang et al., 2023b) |
| Asymmetric HAD hydrogel [HA-g-(AEMA-DA)] | iPSC-CM Exos | Rat cardiac surgery model | 100 μl, |  | Reduced oxidative stress/pericardial adhesion; sustained exosome release | (Wang et al., 2023a) |
| Bilayered cardiac patch (PUAO-CPO-Collagen) | ADSC-Exos | Rat MI model | injected into epicardium directly |  | Enhance cardiac repair; reduced scar formation; enhanced angiogenesis; decreased oxidative stress | (Shiekh et al., 2022) |
| Alginate hydrogel | MSC-Exos | MI model | Patch implantation |  | Reduced apoptosis; promoted macrophage polarization; improved long-term cardiac function | (Lv et al., 2019) |
| Alginate/fibrin hydrogel + AuNPs/CAT (oxygen-generating) | MSC-Exos | Rat MI model | 80 μg of sEVs in 100 μL PBS, 100 μL of alginate hydrogel, and 100 μL of alginate hydrogel incorporating 80 μg of sEVs, Injectable hydrogel |  | Reduced LV remodeling; enhanced angiogenesis; decreased apoptosis/necrosis; increased Cx43 | (Xu et al., 2025) |
| Calcium alginate microgels | MSC-Exos | Rat MI model | 50 µL, Injectable hydrogel |  | Improved echocardiography outcomes; improved cardiac biomarker expression | (Banikarimi et al., 2025) |
| Alginate/collagen/calcium gluconate hydrogel | ADSC-Exos | Female rat MI model | Microgel encapsulation |  | Long-term exosome retention; homogeneous release; reduced off-target accumulation | (Gil-Cabrerizo et al., 2022) |
| Self-assembled peptide amphiphiles (PA) | Xenogeneic hUCMSC-Exos | Rat MI model (28 days) | 30 μL, Injectable hydrogel |  | Reduced fibrosis; down-regulation of TGF-β1; decreased apoptosis; increased CD31+ cells; reduction in CD68-positive inflammatory cells | (Han et al., 2019) |
| Decellularized pericardial scaffold + peptide hydrogel | cATMSC-Exos | Porcine MI model | 20 𝜇g, Peri-infarct injection |  | Improved LVEDV/LVEF/RVEF; reduced scar size/remodeling; modulated inflammation; decreased collagen synthesis and deposition | (Monguió-Tortajada et al., 2022) |
| PUGA-dECM cryogels (polyurethane-gallic acid + dECM) | ADSC-Exos | Rat MI model (8 weeks) | 2 × 107 cATMSCs, Pericardial scaffold |  | Improved cardiac function; reduced fibrosis following patch application; promoted angiogenesis; decreased oxidative stress | (Das et al., 2024) |
| COL-I/Tannic acid hydrogel | MSC-Exos + Se NPs | Rat AMI model | 100 μg, Patch implantation |  | Enhanced cell viability (95% migration); reduced LPS-induced cell apoptosis; improved cardiac function; increased LV wall thickness; reduced infarct size | (Lin et al., 2023) |
| Angiogenin-1 (Ang-1) hydrogel | ISL1-MSC-Exos | MI model | Injectable hydrogel |  | Increased exosome retention; improved anti-apoptotic/proliferative/angiogenic effects | (Hu et al., 2022) |
| RGD-biotin hydrogel | HIF-1α-MSC-Exos | Rat MI model | Injectable hydrogel |  | Reduced caspase-3/7 activity; enhanced lumen formation; improved cardiac function; decreased collagen I deposition | (Wang et al., 2021) |

PUAO-CPO-Collagen: nanofibrous bi-layered cardiac patch, LVEDV: left ventricular end-diastolic volume, LVEF: left ventricular ejection fraction, RVEF: right ventricular ejection fraction, ISL1: Islet-1, HIF-1 alpha: hypoxia-inducible factor 1-alpha, COL-I: collagen type I, TA: tannic acid.

BANIKARIMI, S. P., MELLATI, A., ABASI, M., SOLEIMANI, M., GHIASS, M. A., TAFTI, S. H. A., BOROUMAND, S. & HASANZADEH, E. 2025. Cardiac tissue regeneration by microfluidic generated cardiac cell-laden calcium alginate microgels and mesenchymal stem cell extracted exosomes on myocardial infarction model. *International Journal of Biological Macromolecules,* 292**,** 139247.

DAS, A., NIKHIL, A., SHIEKH, P. A., YADAV, B., JAGAVELU, K. & KUMAR, A. 2024. Ameliorating impaired cardiac function in myocardial infarction using exosome-loaded gallic-acid-containing polyurethane scaffolds. *Bioactive Materials,* 33**,** 324-340.

GIL-CABRERIZO, P., SALUDAS, L., PRÓSPER, F., ABIZANDA, G., DE ANLEO, M. E.-G., RUIZ-VILLALBA, A., GARBAYO, E. & BLANCO-PRIETO, M. J. 2022. Development of an injectable alginate-collagen hydrogel for cardiac delivery of extracellular vesicles. *International Journal of Pharmaceutics,* 629**,** 122356.

HAN, C., ZHOU, J., LIANG, C., LIU, B., PAN, X., ZHANG, Y., WANG, Y., YAN, B., XIE, W. & LIU, F. 2019. Human umbilical cord mesenchymal stem cell derived exosomes encapsulated in functional peptide hydrogels promote cardiac repair. *Biomaterials science,* 7**,** 2920-2933.

HU, X., NING, X., ZHAO, Q., ZHANG, Z., ZHANG, C., XIE, M., HUANG, W., CAI, Y., XIANG, Q. & OU, C. 2022. Islet-1 mesenchymal stem cells-derived exosome-incorporated angiogenin-1 hydrogel for enhanced acute myocardial infarction therapy. *ACS applied materials & interfaces,* 14**,** 36289-36303.

LIN, S., ZHU, Y., HU, T., WANG, K. & CHEN, X. 2023. Novel design of nano-selenium loaded injectable hydrogel combined with mesenchymal stem cells-derived exosomes improving cardiac repair and nursing care after acute myocardial infarction. *Journal of Drug Delivery Science and Technology,* 87**,** 104711.

LV, K., LI, Q., ZHANG, L., WANG, Y., ZHONG, Z., ZHAO, J., LIN, X., WANG, J., ZHU, K. & XIAO, C. 2019. Incorporation of small extracellular vesicles in sodium alginate hydrogel as a novel therapeutic strategy for myocardial infarction. *Theranostics,* 9**,** 7403.

MONGUIÓ-TORTAJADA, M., PRAT-VIDAL, C., MARTÍNEZ-FALGUERA, D., TEIS, A., SOLER-BOTIJA, C., COURAGEUX, Y., MUNIZAGA-LARROUDÉ, M., MORON-FONT, M., BAYES-GENIS, A. & BORRÀS, F. E. 2022. Acellular cardiac scaffolds enriched with MSC-derived extracellular vesicles limit ventricular remodelling and exert local and systemic immunomodulation in a myocardial infarction porcine model. *Theranostics,* 12**,** 4656.

SHIEKH, P. A., MOHAMMED, S. A., GUPTA, S., DAS, A., MEGHWANI, H., MAULIK, S. K., BANERJEE, S. K. & KUMAR, A. 2022. Oxygen releasing and antioxidant breathing cardiac patch delivering exosomes promotes heart repair after myocardial infarction. *Chemical Engineering Journal,* 428**,** 132490.

WANG, L., CHEN, P., PAN, Y., WANG, Z., XU, J., WU, X., YANG, Q., LONG, M., LIU, S. & HUANG, W. 2023a. Injectable photocurable Janus hydrogel delivering hiPSC cardiomyocyte-derived exosome for post–heart surgery adhesion reduction. *Science Advances,* 9**,** eadh1753.

WANG, Q., ZHANG, L., SUN, Z., CHI, B., ZOU, A., MAO, L., XIONG, X., JIANG, J., SUN, L. & ZHU, W. 2021. HIF-1α overexpression in mesenchymal stem cell-derived exosome-encapsulated arginine-glycine-aspartate (RGD) hydrogels boost therapeutic efficacy of cardiac repair after myocardial infarction. *Materials Today Bio,* 12**,** 100171.

WANG, Y., WANG, J., LIU, C., LI, J., LU, K., YU, Q., ZHANG, Y. & SHEN, Z. 2023b. Injectable decellularized extracellular matrix hydrogel loaded with exosomes encapsulating curcumin for prevention of cardiac fibrosis after myocardial infarction. *Journal of Materials Science & Technology,* 167**,** 50-58.

XU, Z., HONG, W., MO, Y., SHU, F., LIU, Y., CHENG, Y., TAN, N. & JIANG, L. 2025. Stem cells derived exosome laden oxygen generating hydrogel composites with good electrical conductivity for the tissue-repairing process of post-myocardial infarction. *Journal of Nanobiotechnology,* 23**,** 213.

YUAN, J., YANG, H., LIU, C., SHAO, L., ZHANG, H., LU, K., WANG, J., WANG, Y., YU, Q. & ZHANG, Y. 2023. Microneedle patch loaded with exosomes containing microRNA‐29b prevents cardiac fibrosis after myocardial infarction. *Advanced healthcare materials,* 12**,** 2202959.
